# Surface Hopping Dynamics with the Frenkel Exciton Model in a Semiempirical Framework

**DOI:** 10.1021/acs.jctc.1c00942

**Published:** 2021-11-29

**Authors:** Eduarda Sangiogo Gil, Giovanni Granucci, Maurizio Persico

**Affiliations:** Dipartimento di Chimica e Chimica Industriale, University of Pisa, via Moruzzi 13, 56124 Pisa, Italy

## Abstract

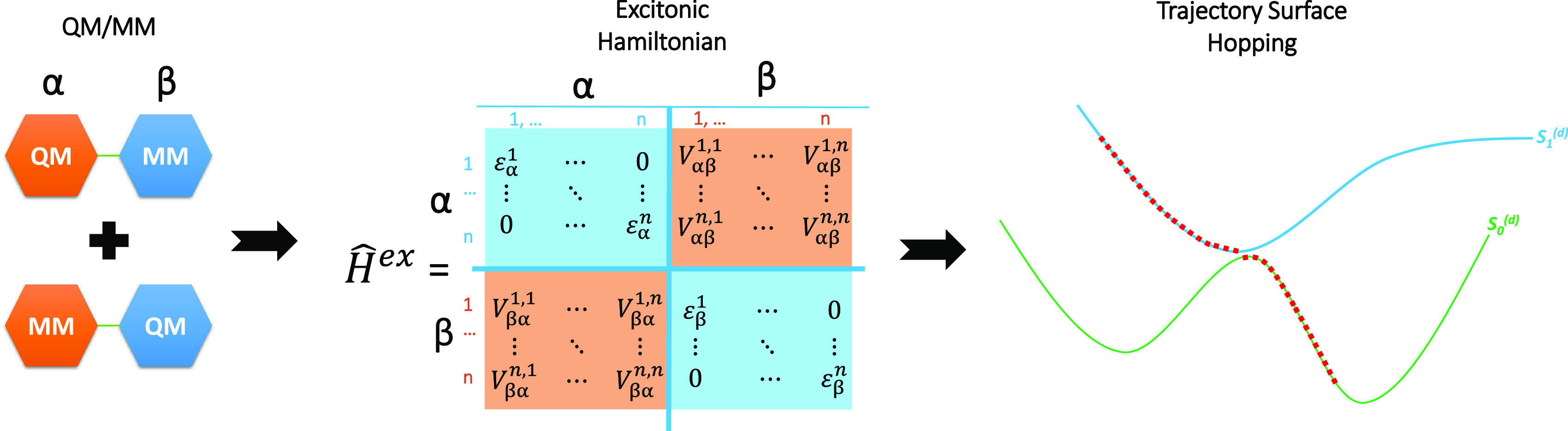

We present an implementation of the Frenkel exciton model in the framework of the semiempirical floating occupation molecular orbitals-configuration interaction (FOMO-CI) electronic structure method, aimed at simulating the dynamics of multichromophoric systems, in which excitation energy transfer can occur, by a very efficient approach. The nonadiabatic molecular dynamics is here dealt with by the surface hopping method, but the implementation we proposed is compatible with other dynamical approaches. The exciton coupling is computed either exactly, within the semiempirical approximation considered, or by resorting to transition atomic charges. The validation of our implementation is carried out on the *trans*-azobenzeno-2S-phane (2S-TTABP), formed by two azobenzene units held together by sulfur bridges, taken as a minimal model of multichromophoric systems, in which both strong and weak exciton couplings are present.

## 1 Introduction

The electronic excitation energy transfer (EET) is a fundamental process, in which electronic excitation is transferred from a donor fragment to an acceptor. This process normally starts with a chromophore (the donor) being optically excited, and then the excitation is transferred to a nearby acceptor. EET is one of the most important photophysical processes in biological and synthetic systems. Probably, the most studied example of the former is the photosynthetic process.^1−3^ In synthetic systems, EET is relevant in photovoltaic cells,^4,5^ photochemical switches,^6,7^ organic optoelectronic devices,^8^ etc.

The study of EET and other aspects of nonadiabatic dynamics in multichromophoric systems calls for employing some sort of “divide and conquer” strategy. In this respect, one of the most successful schemes is offered by the Frenkel exciton model, where the electronic excited states of the multichromophoric system are represented by linear combinations of localized excitations. In this way, an approximate quantum chemical description of the system is obtained evaluating the transition energies associated with the localized excitations (the so-called “site energies”) and the couplings between them. These quantities can be inserted into a purely electronic model Hamiltonian, and the EET rates can be obtained by a variety of approaches.^9^ Alternatively, the Frenkel exciton model can be employed to perform explicit full-dimensional simulations of the nonadiabatic dynamics of the multichromophoric system.^10^ In this respect, a number of implementations have been proposed, all resorting to trajectory-based mixed quantum-classical schemes for molecular dynamics.^11−13^ More in general, an overview of approaches devoted to the modeling of nonadiabatic molecular dynamics in large systems, possibly multichromophoric, can be found in ref (14).

The coupling between excited states localized on different chromophores can be expressed as a sum of Coulomb and exchange terms.^15^ When the electronic states considered belong to the same spin multiplicity, and the two chromophores are not too close, the Coulomb term dominates, so that the exchange interaction is usually neglected. Besides the exact determination of the Coulomb integral,^16^ several approximate computational schemes have been proposed, for instance, those based on transition multipoles or transition atomic charges, fitting the transition density.^13,17,18^ The main problem in this context is the accurate evaluation of the analytical gradient of the exciton coupling, needed to perform molecular dynamics simulations.^13,19^ More challenging, in multichromophoric systems, is the choice of the quantum chemistry method for the computation of site energies and wavefunctions. In this respect, time-dependent density-functional theory (TDDFT) is computationally affordable but suffers from the problems of single reference methods, preventing, for example, a proper description of the decay to the ground state. With multireference methods like CASSCF, the size of the active space which can be afforded is often too small to yield reliable results.

A valid alternative, which may represent a good compromise between accuracy and computational effort in large systems such as multichromophoric assemblies, is offered by semiempirical methods. In this work, we present an implementation of the Frenkel exciton model, which employs the floating occupation molecular orbital-configuration interaction (FOMO-CI) method,^20^ in a semiempirical framework, to evaluate the relevant electronic wavefunctions and site energies. The exciton coupling (Coulomb) is evaluated either exactly, within the semiempirical formalism adopted, or resorting to transition atomic charges. The molecular dynamics simulation is performed according to the surface hopping (SH) scheme, using the local diabatization (LD) algorithm for the numerical integration of the time-dependent Schrödinger equation for the electrons.^21^ It has been noticed that, in multichromophoric systems, crossings between (almost) noninteracting electronic states (the so-called “trivial unavoided crossings”) are common during the dynamics, and this may cause problems in standard implementations of the surface hopping scheme.^22^ In this respect, the LD algorithm, being exempt by construction from problems related to trivial crossings,^10,23^ is particularly well suited for the study of the dynamics of multichromophoric systems. As a test case, we considered the *trans*-azobenzeno-2S-phane (2S-TTABP), in which two azobenzene units are connected by −CH_2_-S-CH_2_– bridges, which represents a minimal example of the multichromophoric system. The simulations of the excited-state dynamics of 2S-TTABP performed with the exciton model are compared with standard surface hopping calculations, in which the electronic states are obtained by quantum chemistry calculations on the whole molecule. The paper is organized as follows: at first, we describe our implementation of the exciton model in the semiempirical framework. Then, we report the results obtained applying the method to the simulation of excited-state dynamics of 2S-TTABP. To make the comparison with the standard “supermolecule” approach easier, an analysis in terms of localized diabatic states is performed for the latter.

## 2 Method

We consider here a system formed by a number *N*_C_ of chromophores, also designated as monomers in the following. It could be a molecular or a supramolecular system, or an assembly of molecules. In the Frenkel exciton model, the electronic Hamiltonian for the system considered is approximated as
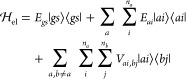
1where |*gs*⟩ is the electronic ground state, with its energy *E_gs_*, while |*ai*⟩ represents the *i*th electronic excited state localized on chromophore *a*, and *n*_*a*_ is the number of excitons |*ai*⟩ considered. The quantity *E*_*ai*_ is the corresponding energy, and the difference Δ*E*_*ai*_ = *E*_*ai*_ – *E*_*gs*_ is the so-called “site energy”. The real quantities *V*_*ai*,*bj*_ = *V*_*bj*,*ai*_ are the excitonic couplings. The set of quasi-diabatic wavefunctions |*ai*⟩ will be referred to as the excitonic basis. These wavefunctions are quasi-diabatic as they retain their character of excitation localized on a given chromophore, but the nature of the excited state *i* within chromophore *a* may change; in fact, the wavefunction |*ai*⟩ is determined as the antisymmetrized product of the *i*th excited adiabatic state of chromophore *a* with the adiabatic ground states of the other monomers (see below). As a consequence, the electronic coupling *V*_*ai*,*aj*_ between two excitonic states belonging to the same chromophore is zero. In the present implementation, the excitonic wavefunctions are obtained according to the following procedure. First, a quantum chemistry calculation is performed for chromophore *a* separately, taking into account the interaction with the other chromophores and the environment by a quantum mechanics/molecular mechanics (QM/MM) scheme with electrostatic embedding. We determine in this way the wavefunctions φ_*i*_^(a)^ and the energies *E*_*i*_^(a)^. *N*_C_ calculations are required, one for each of the chromophores considered. In the semiempirical framework adopted here, the molecular orbitals (MOs) obtained from the QM/MM calculations on different chromophores are orthogonal by construction, as they are built with distinct sets of orthogonal atomic orbitals (AOs). The ground state |*gs*⟩ and the excitonic state |*ai*⟩ are then obtained as antisymmetrized products of the single chromophore wavefunctions
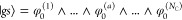
2
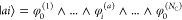
3and form an orthogonal set, according to the above remark on the molecular orbitals. The states φ_*i*_^(a)^ can be taken as McWeeny’s group functions with strong orthogonality conditions.^15,24^ The site energies are obtained simply as Δ*E*_*ai*_ = *E*_*i*_^(a)^ – *E*_0_^(a)^, while the ground state energy *E*_*gs*_ is defined in this way
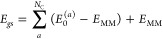
4where *E*_MM_ is the MM energy of the full system. In eq 4, *E*_MM_ is subtracted from each of the QM/MM energies *E*_0_^(a)^ to erase the unwanted contribution of the MM part. However, in this way, the MM energy of chromophore *a* is also subtracted, which is compensated by the last term of eq 4. Let us consider, for example, just two chromophores (*a* and *b*), and express the QM/MM ground-state energy of a monomer as *E*_0_^(a)^ = *E* (QM, *a*) + *E* (MM, *b*) + *E* (QM/MM, *ab*), where the last term is the interaction energy between the QM monomer *a* and the MM monomer *b*. Our definition for the ground-state energy gives therefore *E*_*gs*_ = *E* (QM, *a*) + *E* (QM, *b*) + *E* (QM/MM, *ab*) + *E* (QM/MM, *ba*) – *E* (MM, *ab*), where the last term compensates for double counting of the *a*–*b* interaction. A more general assessment of the implications of eq 4 is shown in the Supporting Information.

Assuming that all of the electronic states considered belong to the same spin manifold and that the distance between the chromophores is large enough to neglect the exchange interaction, the coupling terms *V*_*ai*,*bj*_ between excitonic states |*ai*⟩ and |*bj*⟩ can be approximated by the Coulomb integral^15^ (in atomic units)
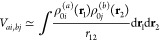
5where ρ_0*i*_^(a)^ is the transition density for the two states φ_0_^(a)^ and φ_*i*_^(a)^ (and similarly for ρ_0*j*_^(b)^). We notice here that, within the semiempirical NDO approximation, the exchange contribution to *V*_*ai*,*bj*_ actually vanishes. Although its evaluation in a semiempirical framework could be attempted on the model of the one-electron two-center integrals, it would require a careful parametrization of two-electron integrals, which was beyond the aim of the present work. In a semiempirical context, the Coulomb integral can be obtained as
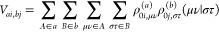
6where *A* is an atom of chromophore *a*, μ and ν are atomic orbitals belonging to *A*, and ρ_0*i*,μν_^(a)^ is an element of the transition density matrix, expressed in the AO basis, for states 0 and *i* of chromophore *a*. The symbol (μν|στ) represents the two-electron repulsion integral in Mulliken notation. Thanks to the NDO approximation, only the integrals with μ and ν belonging to the same atom (as well as σ and τ) have to be evaluated, so reducing considerably the computational burden with respect to an ab initio calculation. However, the number of Coulomb integrals scales as the square of the number of chromophores. Therefore, for large systems, the determination of the *V*_*ai*,*bj*_ couplings may constitute a computational bottleneck. Several approximated approaches^13^ have been proposed and used to evaluate the interchromophore Coulomb integral of eq 5. Here, we consider the method, which consists in approximating, for each monomer, the transition density ρ_0*i*_^(a)^ by a set of “transition” atomic charges **q**_*ai*_. The coupling term *V*_*ai*,*bj*_ is then immediately obtained as the electrostatic interaction of the two sets of atomic charges
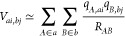
7where *A* and *B* are atoms belonging to monomers *a* and *b*, respectively. The transition atomic charges **q**_*ai*_ are obtained by the TrESP method,^17,18^ which consists in fitting the electrostatic potential arising from the transition density ρ_0*i*_^(a)^.

The diagonalization of the excitonic Hamiltonian  yields the electronic adiabatic wavefunctions |*gs*⟩ and |*K*⟩, with the corresponding energies *E*_*gs*_ and *E*_*K*_. |*gs*⟩ is, by construction, an eigenstate of , while
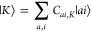
8where the orthogonal matrix **C**, collecting the coefficients *C*_*ai*,*K*_, diagonalizes the excitonic block of . The adiabatic basis is employed in the surface hopping (SH) dynamics simulations, as SH works best in the adiabatic representation.^25^ In fact, at variance with the diabatic couplings (the *V*_*ai*,*bj*_ in the present context), the nonadiabatic couplings are well localized in near degeneracy regions, which allows to minimize the number of hoppings and the extent of the velocity rescaling after a hop. Moreover, no special care is needed to include the superexchange effect, when working in the adiabatic representation.^5,26^ Computation of the gradient ∇*E*_*K*_ of the adiabatic energies with respect to nuclear coordinates is requested to run SH trajectories. Taking into account that the derivatives of the variationally optimized coefficients *C*_*ai*,*K*_ give a null contribution to the gradient, we have
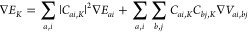
9According to the above definitions, the quantities ∇*E*_*gs*_ and ∇*E*_*ai*_ are obtained as algebraic sums of standard QM/MM and MM gradients. When using the transition atomic charges approximation, eq 7, the computation of the derivatives ∇*V*_*ai*,*bj*_ becomes straightforward by making the simplifying assumption of neglecting the dependence of the transition charges **q**_*ai*_ on the nuclear coordinates.^11^ If instead the Coulomb coupling terms are computed exactly by eq 6, we perform the corresponding approximation of evaluating ∇*V*_*ai*,*bj*_, considering only the static contribution to the derivative (i.e., only the gradient of the two-electron integrals in the AO basis is evaluated, while the gradient of the density matrices is neglected).

We adopt the local diabatization (LD) formalism^21,23,25,27^ for the integration of the electronic time-dependent Schrödinger equation in the surface hopping scheme. It requires the evaluation of the wavefunction overlaps
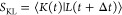
10at each time step along the nuclear trajectory. The *S*_KL_ is obtained by first computing the overlaps in the excitonic basis and then applying the transformation matrix **C**(*t*) of eq 8 to the bra and **C**(*t* + Δ*t*) to the ket. Also, notice that the overlaps involving |*gs*⟩, which allow for the decay of the system to the ground state, have to be considered. Taking into account the orthogonality of MOs belonging to different chromophores, the overlaps between excitonic functions simplify to
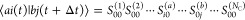
11where *S*_*ij*_^(a)^ = ⟨φ_*i*_^(a)^(*t*)|φ_*j*_^(a)^(*t* + Δ*t*)⟩ are the single chromophore overlaps, and *S*_*i*0_^(a)^ (and/or *S*_0*j*_^(b)^) has to be replaced with *S*_00_^(a)^ (and/or *S*_00_^(b)^) if ⟨*ai*| (and/or |*bj*⟩) of the LHS is replaced with the ground state. The computation of many overlaps, very expensive in an ab initio scheme, is viable in the semiempirical framework. We emphasize here that the LD algorithm is particularly suited to be employed in the present context. In fact, it is exempt from the so-called “trivial crossing” problem,^22^ which is likely to be present in a multichromophoric system, where the diabatic coupling between distant chromophores may easily become vanishingly small. Moreover, no special care is needed for the signs of the couplings *V*_*ai*,*bj*_, which are automatically accounted for in the LD scheme.

Our implementation is as follows: the SH nonadiabatic dynamics simulations are performed exploiting the Newton-X package,^28^ modified to run exciton dynamics calculations. The QM/MM quantum chemistry calculations needed to run the SH trajectories on the fly are done with our modified version of the semiempirical program MOPAC2002,^29^ interfaced with the molecular mechanics TINKER^30^ program package. In the following, we will label as exact Coulomb (EC), the exciton scheme in which the Coulomb couplings are evaluated exactly using eq 6, and transition charges (TC), the exciton model where the couplings are approximated resorting to TrEsp charges.

## 3 Validation

**Figure 1:**
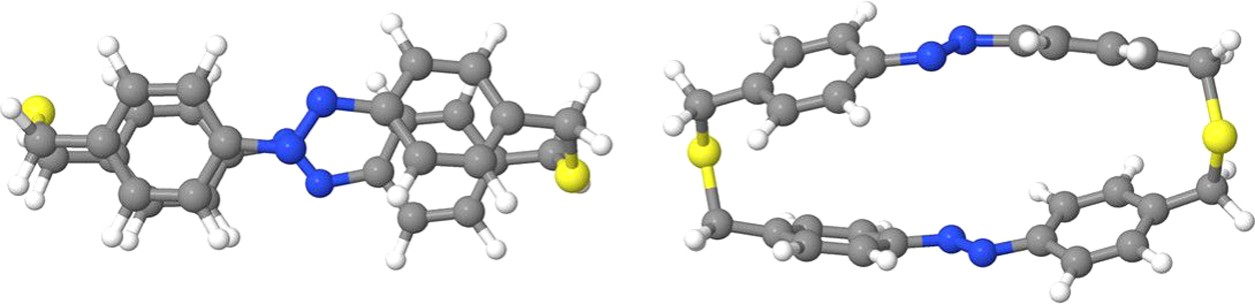
Lateral and top views of 2S-TTABP.

As a test case, we considered the photoisomerization dynamics of a molecular system formed by only two chromophores: the *trans*-azobenzeno-2S-phane (2S-TTABP); see Figure 1. The two monomeric units are represented in this case by the two azobenzene moieties of 2S-TTABP. We considered both nπ* and ππ* localized excitations. While the former are very weakly coupled, the latter show larger excitonic couplings, in agreement with the transition dipole moments of the monomers. For comparison purposes, the simulation of the photoisomerization of 2S-TTABP has been performed using both the exciton model and the standard approach (hereafter, labeled as the “full-QM”), where the electronic states are obtained from quantum chemistry calculations on the whole molecule.

### 3.1 Computational Details

Electronic wavefunctions and energies have been computed using the FOMO-CI method with a semiempirical AM1 Hamiltonian, replacing the standard set of AM1 parameters with another one, previously reoptimized for azobenzene by us.^20^ For the QM/MM calculations, a truncated CI within an active space of 13 MOs and 14 electrons was considered^20^ for a total of 94 Slater determinants. The full-QM calculations were performed considering a CAS-CI space obtained from an active space of 8 MOs and 12 electrons, which included two nonbonding orbitals (one for each azobenzene moiety) and four π and two π* orbitals. To avoid intrusion in the active space of the unwanted nonbonding sulfur orbitals, we adopted the same strategy already employed for azobenzene, consisting in the use of different semiempirical parameters in the SCF and CI calculations. In particular, in the SCF calculations, the parameters *U*_s_ and *U*_p_ of sulfur were decreased, respectively, by 14 and 12 eV with respect to the AM1 values, while the CI calculations use the standard AM1 parameters. The QM/MM calculations were performed using the OPLS force field and with electrostatic embedding.^31^ The partition in a QM and a MM moiety was the same already considered in our previous QM/MM study on 2S-TTABP.^32^

The initial conditions for the nonadiabatic surface hopping simulations were sampled from ground-state thermal trajectories at 300 K. For the excitonic approach, we used the Andersen thermostat^33^ for 30 ps, while for the full-QM calculations, we ran a 50 ps equilibration with the Bussi–Parrinello thermostat.^34^ The use of two different thermostats was dictated by technical reasons. In both methods, the thermalization is obtained by stochastically altering (according to Boltzmann statistics) the nuclear velocities at regular intervals along the classical trajectory. The main difference between the two algorithms is that, while in the Bussi–Parrinello thermostat, all of the nuclear velocities are altered by the same factor, the Andersen thermostat operates independently on each atom. More details on the thermalization can be found in Section S4. The starting conditions (coordinates, velocities, and starting state) for the SH trajectories were selected according to two excitation energy windows, corresponding, respectively, to nπ* and ππ* excitation; see Table 1. The sampling was performed taking into account the radiative (dipole) transition probability, according to the method outlined in ref (25). As expected, after nπ* excitation, *S*_1_^(d)^ and *S*_2_^(d)^ are almost equally populated, while the ππ* excitation populates mostly the bright combination of the two localized excitations *S*_2_*S*_0_ and *S*_0_*S*_2_, which corresponds to *S*_4_^(d)^ in the exciton model and to *S*_5_^(d)^ at the full-QM level (see Section S4). All of the SH calculations were performed with the LD algorithm,^35^ with an integration time step of 0.1 fs (both for the nuclear and for the electronic degrees of freedom). We made use of the overlap-based decoherence correction^35^ (ODC), with Gaussian width σ = 1 au and minimum overlap threshold *S*_min_ = 0.005. The trajectories were stopped after running on the ground state for at least 100 fs.

**Table 1:** SH Simulations for 2S-TTABP: Number of Trajectories, Excitation Energy Windows (eV), Photoisomerization Quantum Yields, Diabatic Lifetimes in ps^a^

method	excitation window	*N*_t_^b^	Φ^c^	τ_1_	τ_2_	*t*_0_
full-QM	nπ* [2.3 eV, 3.3 eV]	278/278	0.29 ± 0.03	0.29		0.06
	ππ* [3.6 eV, 4.8 eV]	389/392	0.28^d^ ±0.02	0.25	0.12	0.08
exciton (EC)	nπ* [2.2 eV, 3.2 eV]	303/306	0.29 ± 0.03	0.54		0.18
	ππ* [3.5 eV, 5.5 eV]	482/482	0.29 ± 0.02	0.50	0.53	0.30
exciton (TC)	nπ* [2.2 eV, 3.2 eV]	304/306	0.31 ± 0.03	0.55		0.18
	ππ* [3.5 eV, 5.5 eV]	479/482	0.28 ± 0.02	0.53	0.50	0.30

a See Sections 3.3 and S5 for the definition of the decay times τ_1_ and τ_2_ and the delay time *t*_0_.

b Number of trajectories we have considered in our analysis/total number of trajectories (a few trajectories are discarded for technical reasons).

c Quantum yield of the *trans* → *cis* photoisomerization. The binomial standard deviation, obtained as , is also shown.

d In this case, the quantum yield has been evaluated discarding the 46 trajectories which, at the end of the simulation, turn out to be trapped in the *TT*_1_ minimum (see Section 3.3).

Hereafter, we will label the electronic adiabatic states as *S*_*n*_ and *S*_*n*_^(d)^, respectively, for the monomer and the full molecule (the superscript *d* stands for dimer). With an obvious notation, the states belonging to the excitonic basis will be indicated as *S*_0_*S*_0_, *S*_1_*S*_0_, *S*_0_*S*_1_, *S*_2_*S*_0_, *S*_0_*S*_2_, *S*_3_*S*_0_, and *S*_0_*S*_3_. To make the comparison with the exciton model easier, the full-QM photodynamics results were analyzed in terms of diabatic states, according to the diabatization scheme recently devised in our group.^36^ In particular, after localization of the active MOs on the two chromophores, a set of reference electronic wavefunctions is built, and the diabatic states are obtained by rotating the adiabatic ones to achieve maximum overlap with the references (see Section S2 in the Supporting Information). In the current work, we have considered the following 13 diabatic states, labeled similarly to the excitonic basis: *S*_0_*S*_0_, the ground state; *S*_0_*S*_1_ and *S*_1_*S*_0_, the localized nπ* excitations on the two chromophores; *S*_0_*S*_2_, *S*_2_*S*_0_, *S*_0_*S*_3_, and *S*_3_*S*_0_, the localized ππ* excitations on the two chromophores; *S*_1_*S*_1_, the combination of two nπ* localized excitations; *TT*_1_, *TT*_2_, and *TT*_3_, the singlet combinations of two localized triplets (shorthands for ^1^(*T*_1_*T*_1_), ^1^(*T*_1_*T*_2_), and ^1^(*T*_2_*T*_1_), respectively); and finally, the charge transfer states *A*^+^*B*^–^ and *A*^–^B^+^. As one can see, this set of diabatic states (with the exception of *TT*_*n*_, *S*_1_*S*_1_, and charge transfer states) is directly comparable with the excitonic states of the EC and TC treatments.

### 3.2 Potential Energy Surfaces (PESs) and Absorption Spectra

**Figure 2:**
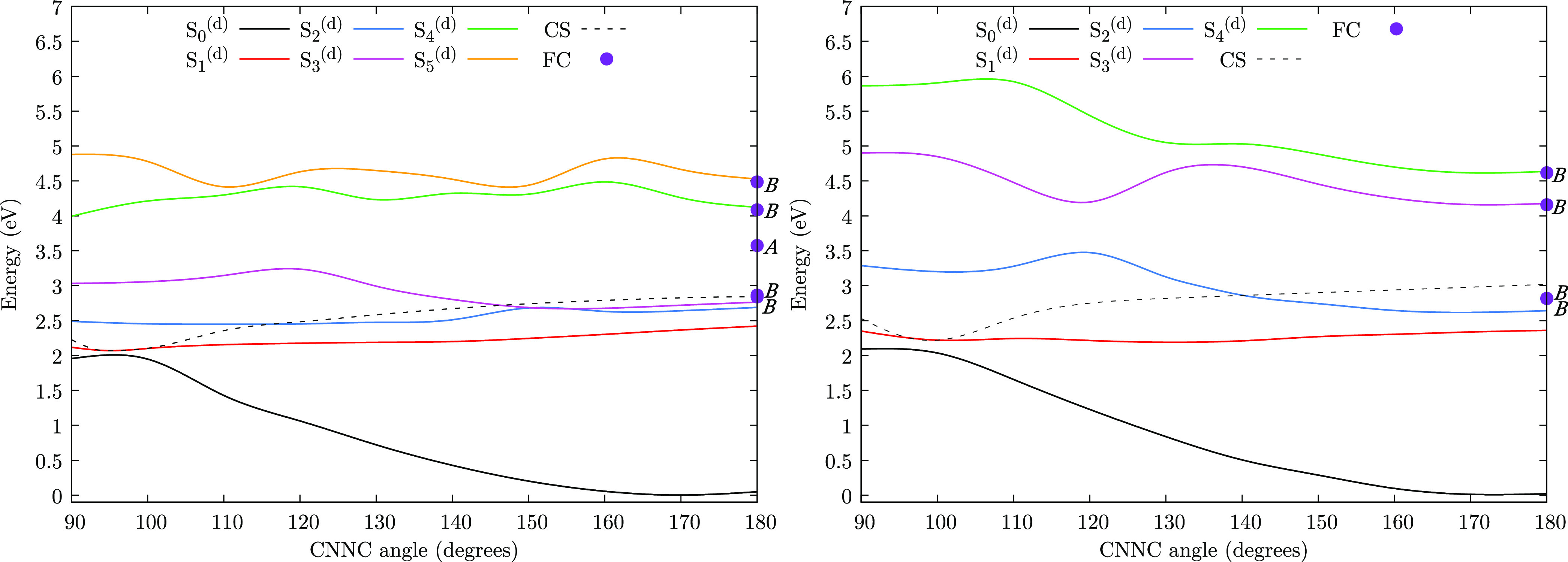
Potential energy curves of the lowest singlet states as functions of one CNNC dihedral. All of the other coordinates have been optimized for each state (except for the last two states shown, which are computed at the *S*_0_^(d)^ optimal geometries). Left panel: full-QM. Right panel: exciton model. Franck–Condon (FC) points are represented as dots, with symmetry labels (*C*_2_ symmetry). The dashed curves represent the *S*_0_^(d)^/*S*_1_^(d)^ crossing seam.

The most important coordinates in the *trans* → *cis* isomerization are of course the CNNC dihedrals. We present therefore in Figure 2 the potential energy curves of the first few singlet states along one of the two CNNC dihedrals. The two excitonic approaches yield very similar PESs; in Figure 2, we show those obtained with the TC scheme (the PESs obtained with the EC approach are shown in the Supporting Information).

At the ground-state equilibrium geometry, the molecule belongs to the *C*_2_ symmetry group, and the *S*_1_^(d)^ and *S*_2_^(d)^ states, both of *B* symmetry, are almost degenerated due to the weak Davydov splitting of nπ* states. In particular, they are found at 2.840 and 2.864 eV above the ground state at the full-QM level, at 2.812 and 2.821 eV at the exciton EC level, and at 2.816 and 2.817 eV at the exciton TC level. When minimizing the energy of *S*_1_^(d)^ or *S*_2_^(d)^, the symmetry is broken, which leads to a removal of the near degeneracy between the two states; see Figure 2. Besides the two nπ* localized excitations, in the left panel of Figure 2, we can also see the singlet combination of the two localized triplets ^3^nπ*, labeled as *TT*_1_, which is only present in the full-QM calculations. In particular, at the Franck–Condon (FC) point, the *S*_3_^(d)^ state (A symmetry), mainly corresponding to *TT*_1_, is found at 3.57 eV above the ground state, which roughly corresponds to twice the energy of the lowest triplet state (1.76 eV). Clearly, the *TT*_1_ state is not included in the excitonic basis. At the FC point, the first two ππ* states are found at 4.086 and 4.486 eV above the ground state at the full-QM level, 4.159 and 4.617 eV at the exciton TC level, and 4.170 and 4.604 eV at the exciton EC level. Therefore, the Davydov splitting of the ππ* states amounts to 0.40 eV at the full-QM level, and it is slightly larger if computed with the exciton model (0.46 and 0.43 eV at the exciton TC and EC levels, respectively).

The full-QM *S*_1_^(d)^ PES shows a steeper slope along the CNNC dihedral if compared to the exciton model. Moreover, at the FC geometry, *S*_1_^(d)^ and *S*_2_^(d)^ are very close in energy to the *S*_1_^(d)^/*S*_0_^(d)^ crossing seam at the full-QM level, while they are well below when considering the exciton model; see Figure 2. Both features point toward a faster nπ* decay dynamics in the full-QM case if compared to the exciton model. The PES for the two upper states, which are *S*_4_^(d)^ and *S*_5_^(d)^ for the full-QM approach and *S*_3_^(d)^ and *S*_4_^(d)^ for the excitonic model, is also shown in Figure 2. The curves computed at the full-QM level (*S*_4_^(d)^ and *S*_5_^(d)^) are significantly different from those calculated with the exciton model (*S*_3_^(d)^ and *S*_4_^(d)^), especially at twisted geometries. This is due to the intrusion of the *TT*_2_ and *TT*_3_ states in the ππ* manifold at the full-QM level. Particularly, *S*_4_^(d)^ and *S*_5_^(d)^ preserve their ππ* character at CNNC > 160°, but *S*_5_^(d)^ acquires a *TT*_2_ character around CNNC = 160°. Then, at 140°, *S*_4_^(d)^ becomes essentially *TT*_2_ and *S*_5_^(d)^ reverts to ππ*, while at lower dihedrals, *S*_4_^(d)^ and *S*_5_^(d)^ correspond to *TT*_2_ and *TT*_3_, respectively. Therefore, the potential energy curves above the nπ* states are poorly described by our exciton approach that does not account for the singlet combinations of two triplets.

**Figure 3:**
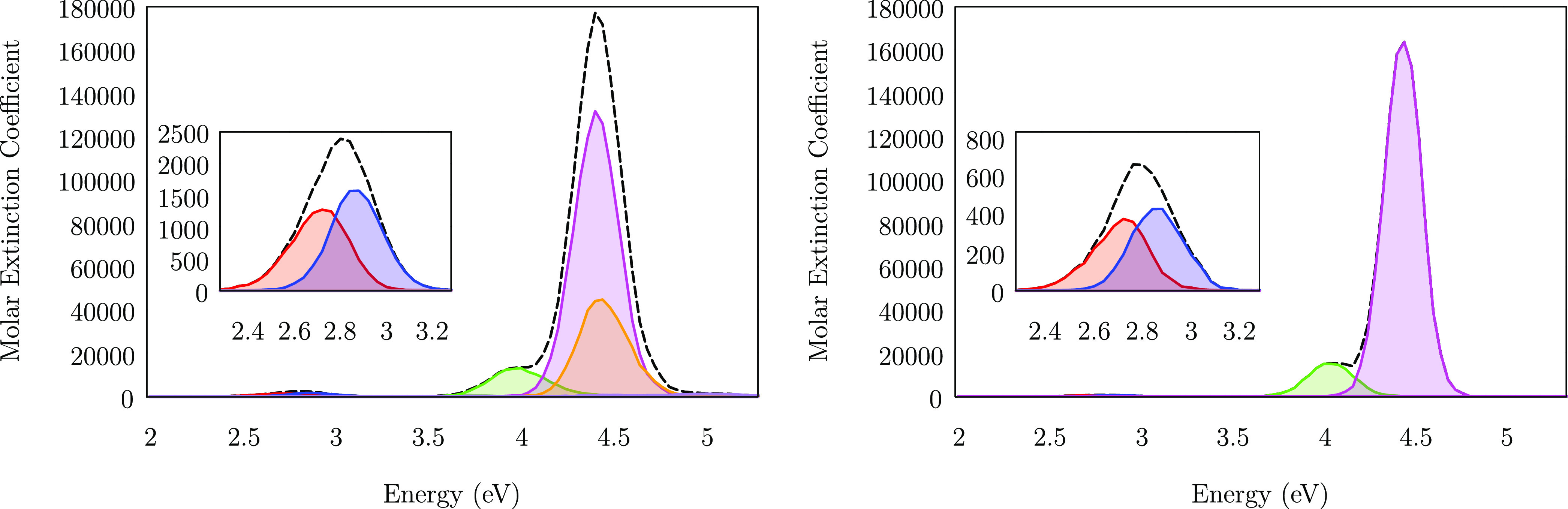
Simulated absorption spectra. Full-QM on the left and exciton TC model on the right. The region of the nπ* band is enlarged in the insets. The contribution of each adiabatic state is shown. The dashed line represents the total spectrum.

Figure 3 presents the computed absorption spectra obtained from the thermalization trajectories with full-QM and exciton TC models. The two excitonic approaches show very similar spectra (see the Supporting information). As observed above, the splitting of the two nπ* sub-bands (see the insets of Figure 3) is not due to the Davydov coupling but rather to the sampling of asymmetrical configurations. The allowed ππ* band presents the characteristic pattern of π-stacked compounds, where the exciton coupling gives rise to a higher lying bright state and a lower lying (almost) dark state.^15^ The energy difference between the maxima of the two ππ* sub-bands amounts to about 0.4 eV with all the three methods, in good agreement with the Davydov splitting obtained at the FC geometry (see above). The experimental spectrum of 2S-TTABP in dichloromethane^37^ shows a weak nπ* band at 2.63 eV and a strong ππ* band, peaked at 3.67 eV, with a shoulder red-shifted to about 0.4 eV with respect to the maximum. Therefore, although in our computed spectra, the ππ* band is significantly blue-shifted with respect to the experimental one, it appears that the Davydov splitting of the ππ* states is reproduced correctly by our semiempirical FOMO-CI calculations.

The interchromophore exchange interaction, besides contributing to the exciton coupling, is a repulsive term, which may be important in the correct assessment of the (ground state) geometrical arrangement of molecular assemblies. In some π-stacked systems, treated at the full-QM semiempirical level, to reproduce correctly the interaction between two monomers, we were forced to add ad hoc Lennard-Jones potentials,^38^ in part to compensate for the neglect of the repulsive intermonomer exchange interaction at the semiempirical NDO level. Considering 2S-TTABP, the good agreement found with the experimental Davydov splitting of the ππ* absorption band is an indication of the correctness of the ground-state equilibrium geometry we obtained at the semiempirical level. Therefore, the exchange interaction is probably not playing an important role in 2S-TTABP (but of course we cannot exclude some error cancellation). This can be due to the fact that the two monomers of 2S-TTABP are not perfectly H-stacked, as can be appreciated from Figure 1 (right panel).

In Table 2, we show the averages and standard deviations of the exciton couplings *V*_*ai*,*bj*_ computed at 300 geometries extracted randomly from the full-QM equilibration trajectory. In particular, we compare the exciton couplings, evaluated by the EC and the TC schemes, with the corresponding diabatic matrix elements obtained from the full-QM approach after diabatization, according to the procedure cited in Section 3.1. As expected, the couplings between ππ* excitons (i.e., ) are significantly higher than the nπ* ones (namely, ), in line with what we have observed about the absorption spectra. In fact, the nπ* couplings oscillate around zero due to conformational changes along the thermalization trajectory, so their averages tend to vanish and only their standard deviations should be taken into consideration. On the contrary, the larger ππ* couplings have well-defined signs, once the diabatic wavefunctions have been assigned their conventional phases. The couplings between nπ* and ππ* excitons, not shown here, present an intermediate behavior between the nπ* and ππ* ones. Overall, the average strengths of the exciton couplings are in good agreement with the corresponding diabatic quantities obtained from the full-QM calculations; see Table 2.

**Table 2:** Electronic Couplings  and  Calculated at the Full-QM, Exciton EC, and Exciton TC Levels^a^

	*V* (nπ*, nπ*)	*V* (ππ*, ππ*)
full-QM	0.2 ± 2.1	180.2 ± 9.4
exciton EC	0.0 ± 1.1	136.7 ± 15.9
exciton TC	0.0 ± 0.6	144.0 ± 16.9

a Presented are averages and standard deviations of the couplings (in meV) computed for 300 geometries sampled randomly from the full-QM thermalization trajectory.

To assess the validity of the TC approach, a detailed comparison of TC couplings with the numerically exact EC ones is reported in Section S3. The nπ* TC couplings show large relative errors with respect to the numerically exact EC couplings. However, the absolute errors are small (less than 0.0015 eV). Concerning the larger ππ* couplings, a systematic deviation of TC values from the EC ones is apparent. In particular, the TC couplings exceed the EC ones by about 0.01 eV, a value which is slowly increasing with the coupling. This phenomenon is probably generated by the partial compenetration of the transition charge clouds of the two chromophores, an effect which cannot be reproduced by atomic charges, which is expected to give increasingly larger deviations for larger couplings. To test this hypothesis, the two −CH_2_-S-CH_2_– linkers present in 2S-TTABP were removed, so as to be able to vary the stack distance between the two azobenzene units. In this way, the coupling between ππ* excitons evaluated at the TC level converges toward the EC value when the stack distance arrives at about 12 Å (see Section S3).

### 3.3 Simulations of the Photodynamics

As mentioned above, both the photodynamics after nπ* excitation and after ππ* excitation were simulated. During the decay to the ground state, one of the two *trans*-azobenzene units of 2S-TTABP may isomerize to *cis*, in which case, the trajectory will be labeled as “reactive”. The *trans* → *cis* photoisomerization quantum yields are shown in Table 1. Overall, the quantum yields obtained with the three methods are very similar among them, with variations well within the error bars. Experimentally, Rau and Lüddecke^39^ measured Φ = 0.24 and 0.21 after nπ* and ππ* excitation, respectively. Therefore, not only are our results able to reproduce correctly the lack of wavelength dependence of the quantum yield in 2S-TTABP (if compared to bare azobenzene, which notoriously violates Kasha’s rule), but our computed values for Φ are also in semiquantitative agreement with the experimental ones.

**Figure 4:**
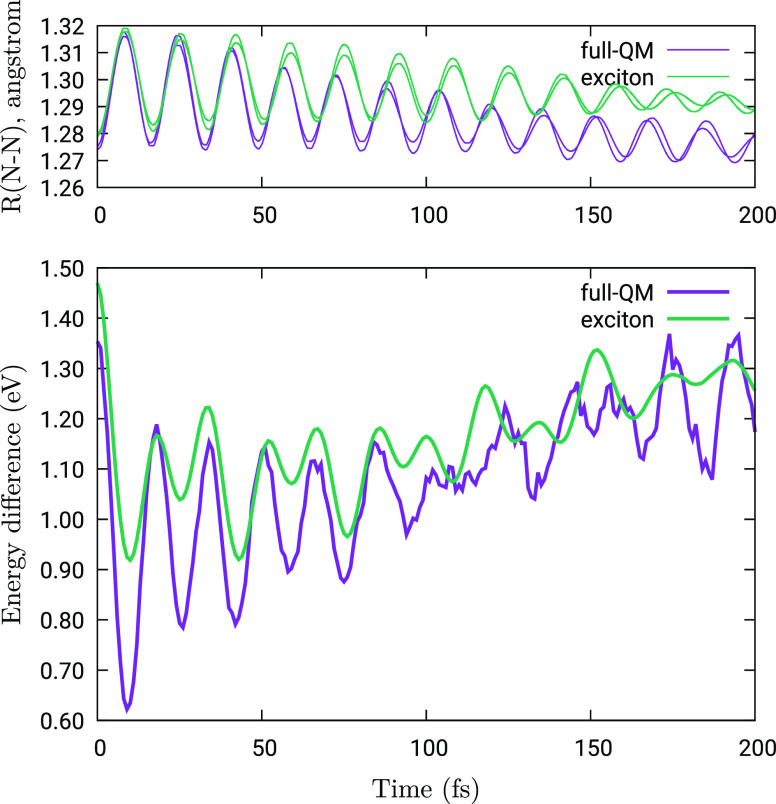
Top panel: the two N–N distances versus time, averaged over the full swarm of trajectories. Both full-QM and exciton TC results are shown. Bottom panel: difference (in eV) between the energy of ππ* states (evaluated as [*E*(*S*_2_*S*_0_) + *E*(*S*_0_*S*_2_)]/2) and the energy of nπ* states (i.e., [*E*(*S*_1_*S*_0_) + *E*(*S*_0_*S*_1_) ]/2), averaged over all trajectories. Both full-QM and exciton TC results are shown.

The lifetimes reported in Table 1 were obtained by fitting the decay of the diabatic populations *P*_nπ*_ = *P*(*S*_1_*S*_0_) + *P*(*S*_0_*S*_1_) and *P*_ππ*_ = ∑_*n*>1_ [*P*(*S*_*n*_*S*_0_) + *P*(*S*_0_*S*_*n*_) ] with two exponential functions, according to a first-order kinetic model for irreversible decay. More in detail, the *P*_ππ*_(*t*) data were fitted with a simple exponential e^–*t*/*τ*_2_^, while the *P*_nπ*_(*t*) function e^–(*t*–*t*_0_)/τ̃_1_^ included the delay time *t*_0_. The overall lifetime of the nπ* states is then τ_1_ = *t*_0_ + τ̃_1_ (see Section S5 of the Supporting information). The two exciton schemes EC and TC are characterized by very similar lifetimes. However, the decay times evaluated with the full-QM approach are noticeably smaller, as can be appreciated from Table 1. This is especially evident for the decay from ππ* states; in fact, the lifetime τ_2_ evaluated with the excitonic model is about 4 times larger than that obtained with the full-QM approach. The *TT*_1_ state (absent in the exciton model calculations) is found in energy between the nπ* and the ππ* states. It is therefore well placed to enhance the decay rate from ππ* states. However, the net population transfer from ππ* states to *TT*_1_ is weak (about 12%), while the net population transfer from *TT*_1_ to other states is vanishing (see below). Actually, the most important decay channel for ππ* states is, by far, both for full-QM and exciton model calculations, the direct transfer to nπ* states. In terms of diabatic (or excitonic) states, this may happen in two ways: either by excitation transfer from one chromophore to the other (i.e., *S*_2_*S*_0_ → *S*_0_*S*_1_ or *S*_0_*S*_2_ → *S*_1_*S*_0_) or by internal conversion within the same monomer (i.e., *S*_2_*S*_0_ → *S*_1_*S*_0_ or *S*_0_*S*_2_ → *S*_0_*S*_1_). Only a very limited number of trajectories follow the former route: 6% in the full-QM simulations, which reduces to about 2.5% in the exciton TC or EC dynamics (see Section S8). However, the lower number of ππ* → nπ* EET in the exciton dynamics cannot account for the slower decay with respect to the full-QM case, which has, therefore, to be attributed to the single-chromophore nonadiabatic dynamics. As the latter is treated in the same way by the full-QM or the exciton models, it appears that the difference in the τ_2_ lifetimes is mainly due to the differences in the PESs. In particular, the ππ* states are closer in energy to the nπ* states in the full-QM calculations. To make this evident, we show in Figure 4 the energy difference between ππ* and nπ* states, averaged over the full swarm of trajectories. Only the first 200 fs of dynamics are considered, as most of the ππ* population decays during that time (see Figure 7). We notice that the ππ*–nπ* energy difference oscillates in phase with the N–N distances. In fact, considering, for example, the full-QM scheme, the average of the two equilibrium N–N bond lengths in 2S-TTABP amounts to about 1.27 Å for both the ground state and *S*_1_^(d)^ (i.e., the lowest nπ* state) and rises to about 1.30 Å for *S*_5_^(d)^ (which corresponds, at transoid geometries, to the bright combination of the *S*_2_*S*_0_ and *S*_0_*S*_2_ππ* states). At short times, the ππ* energies are found to approach the nπ* ones considerably more in the full-QM simulations than in the exciton model ones. As the transition probability between electronic states is strongly dependent on the energy gap, this effect clearly explains the large difference in the τ_2_ lifetimes.

**Figure 5:**
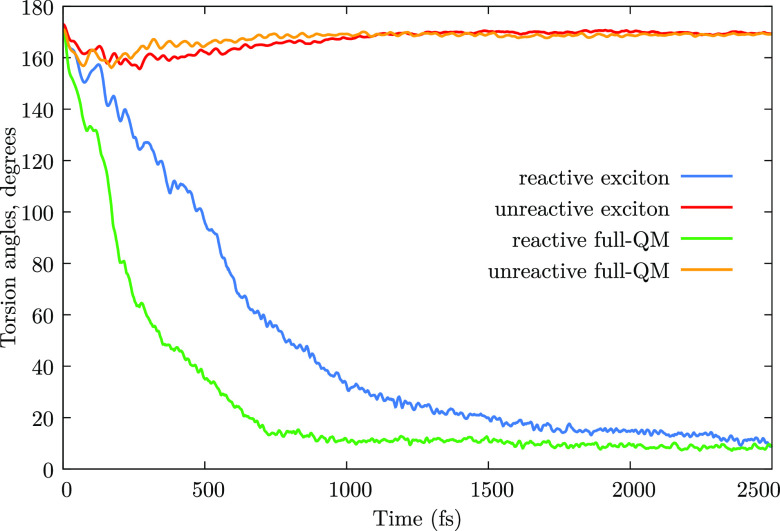
CNNC torsion angles as a function of time, averaged separately for reactive and unreactive trajectories, after nπ* excitation. Presented are results obtained with the full-QM and exciton TC approaches (see the Supporting Information for exciton EC results). Only the CNNC dihedral of the isomerizing monomer is taken into account when averaging over the reactive trajectories, whereas both CNNC dihedrals are averaged for the unreactive trajectories.

The τ_1_ lifetimes, concerning the decay of the nπ* states to the ground state, are slightly longer by exciting to the nπ* states than to the ππ* ones (see Table 1). In both cases, no transitions can occur before a certain degree of twisting has been reached, so as to approach the *S*_0_^(d)^/*S*_1_^(d)^ crossing seam. This is why a delay time elapses before the conversion to the ground state is set off. The decay of the nπ* states populated after a ππ* excitation is slightly faster probably because of the larger vibrational energy, which allows to reach more easily the crossing seam (see Figure 2 and previous work on the photodynamics of azobenzene^40,41^). Larger differences are found by comparing the exciton model simulations with the full-QM ones: the τ_1_ lifetimes in the former case are about twice as large as in the latter. Again, this difference is attributed to the PESs. In fact, as already noticed in Section 3.2, the PES of *S*_1_^(d)^ along the CNNC coordinate, evaluated with the full-QM approach, shows a steeper slope toward the conical intersection located at 90° of torsion, therefore leading to faster dynamics on the *S*_1_^(d)^ PES with respect to the excitonic model schemes. This is confirmed by Figures 5 and S13–S15, where we show the CNNC dihedral as a function of time, averaged separately for reactive and unreactive trajectories. Clearly, the CNNC dihedral of reactive trajectories closes faster in the full-QM approach, so as to wash out oscillations, distinctly present at early times (say, within 250 fs) in the exciton model calculations.

**Figure 6:**
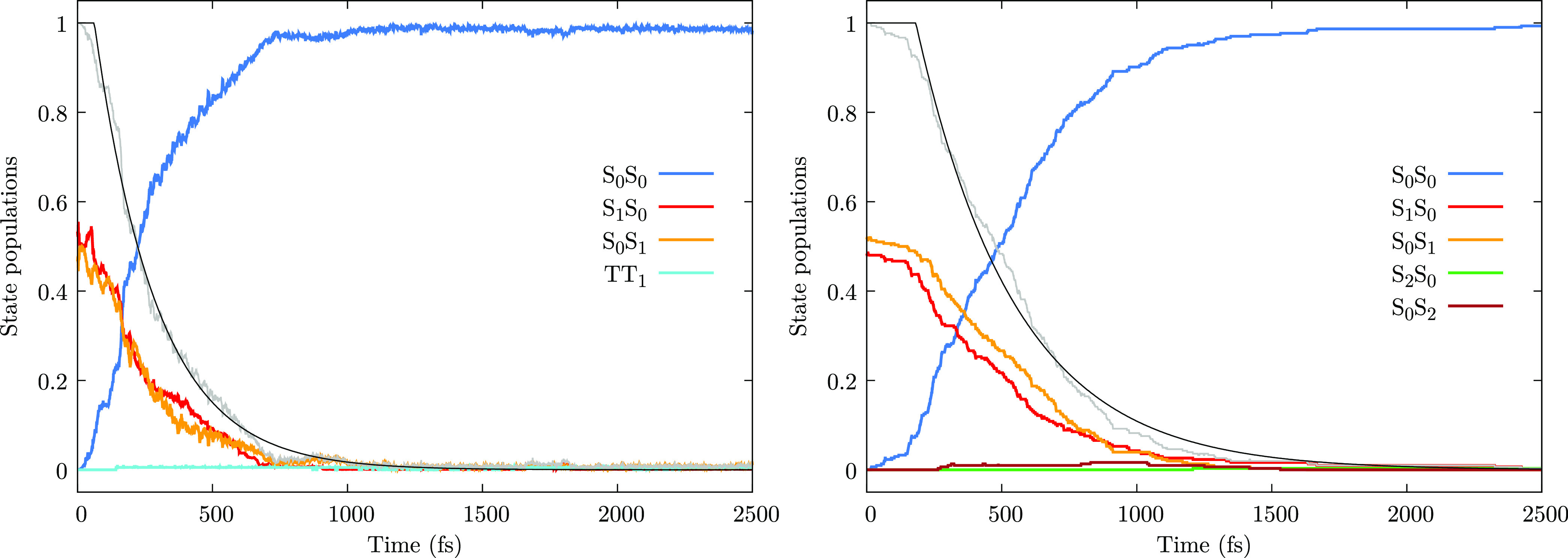
Dynamics after nπ* excitation. Left panel: diabatic state population as functions of time, averaged over all trajectories, for the full-QM approach. Right panel: populations of the excitonic states, averaged over all trajectories, obtained with the exciton TC scheme (see Section S7 for the results obtained with the EC scheme, very similar to the TC ones). Gray line: total nπ* population, *P*_nπ*_; black line: fit of *P*_nπ*_ according to the kinetic model of Section S5.

In Figure 6, we show the population of the diabatic (or excitonic) states, averaged over all trajectories, as functions of time, after nπ* excitation. The corresponding adiabatic populations are shown in Section S6. Apart from the difference in the decay rate discussed above, the full-QM approach and the exciton scheme show a very similar behavior. In particular, at the beginning of the simulation, *S*_1_^(d)^ and *S*_2_^(d)^ are almost equally populated. Very rapidly, within the first 10 fs, all of the population is transferred to *S*_1_^(d)^, which localizes to either *S*_1_*S*_0_ or *S*_0_*S*_1_. During the dynamics, until the decay to the ground state, *S*_1_^(d)^ keeps its localized character. In other words, as expected considering the weak nπ* couplings, there is no EET.

**Figure 7:**
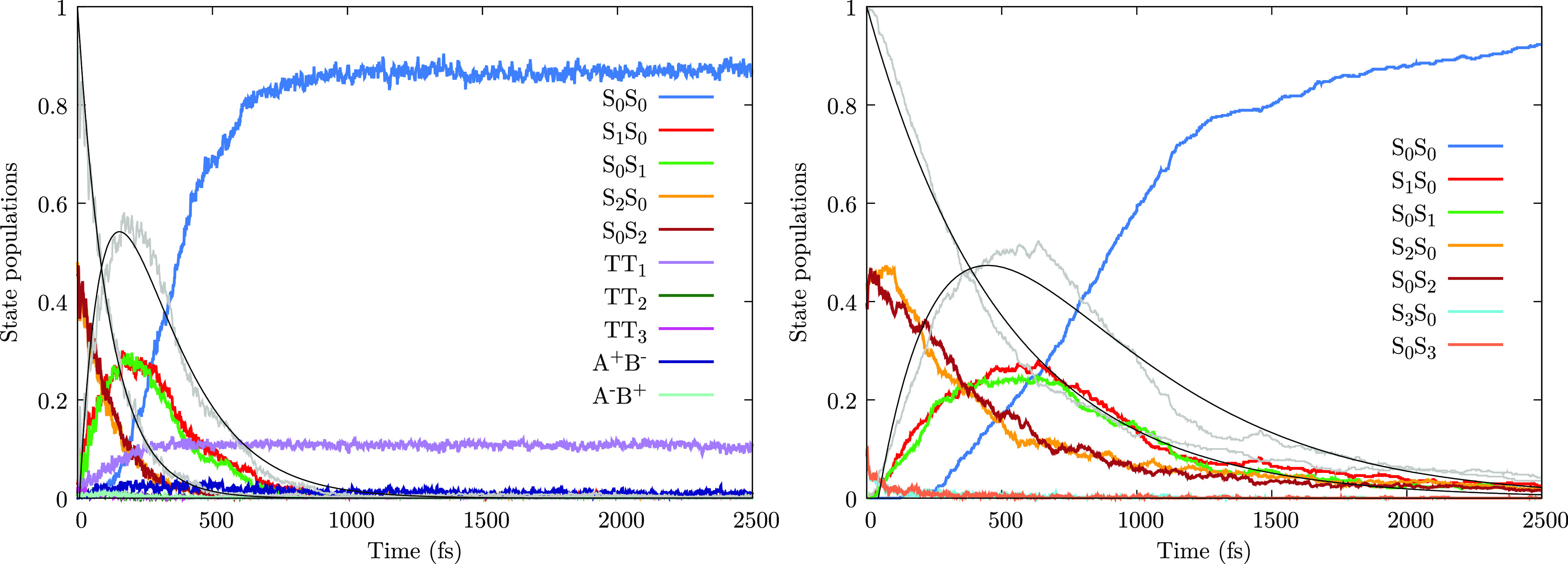
Dynamics after ππ* excitation. Left panel: diabatic state population as functions of time, averaged over all trajectories, for the full-QM approach. Right panel: populations of the excitonic states, averaged over all trajectories, obtained with the exciton TC scheme (see Section S7 for the results obtained with the EC scheme, very similar to the TC ones). The nπ* and ππ* populations (*P*_nπ*_ and *P*_ππ*_; see the text) are shown with gray lines. Black lines: fit of *P*_nπ*_ and *P*_ππ*_ according to the kinetic model of Section S5.

The diabatic states populations after ππ* excitation are shown in Figure 7. In this case, at variance with nπ* dynamics, there is a noticeable qualitative difference between full-QM and exciton model calculations. In fact, the *TT*_1_ state, absent in the exciton model, acquires, in the full-QM simulations, a non-negligible population (12%). However, as noticed above, the decay to the nπ* states is hardly influenced by the presence of the *TT*_1_ state, as the population transferred to *TT*_1_ gets stuck in it, within the first 2.5 ps, see Figure 7 and Section S8. The trajectories trapped in *TT*_1_ oscillate around the *TT*_1_ minimum, which is characterized by both CNNC dihedrals close to 93°. At that geometry, the *TT*_1_ state actually corresponds to *S*_0_^(d)^, and such a local minimum of the ground state is found 2.01 eV above the *S*_0_^(d)^ all-trans minimum of Figure 1. The search for the transition state connecting the two minima on the ground state led to a *S*_0_^(d)^/*S*_1_^(d)^ conical intersection, well described as a crossing between *TT*_1_ and *S*_0_*S*_0_. Such feature is close in energy to the *TT*_1_ minimum (only 0.22 eV above it) and has the two CNNC dihedrals presenting a value of 123°. It is therefore likely that the trajectories escaping from the *TT*_1_ local minimum revert back to the all-trans configuration. In the evaluation of the photoisomerization quantum yield for the full-QM simulations, we discarded the trajectories trapped in the *TT*_1_ minimum. Assuming that all of those trajectories would revert back to the all-trans isomer (given the transoid geometry of the transition state referred above), the full-QM photoisomerization quantum yield after ππ* excitation would show a modest decrease from 0.28 to 0.25.

Excitation energy transfer is observed after ππ* excitation. In fact, a very large number of transitions were observed between *S*_2_*S*_0_ and *S*_0_*S*_2_ excitons, amounting to 12.3 and 13.7 transitions per trajectory with the EC and TC schemes, respectively. This difference is somehow expected, taking into account the larger ππ* couplings computed with the TC approach, see Table 2. With the full-QM approach, we obtained an average of 12.6 transitions per trajectory between the diabatic states *S*_2_*S*_0_ and *S*_0_*S*_2_, in very good agreement with the exciton model calculations. However, this may result from a compensation of opposite effects: on the one hand, the diabatic full-QM ππ* couplings are larger than the corresponding excitonic ones, and on the other hand, the ππ* states remain populated for a shorter time in the full-QM simulations, thus decreasing the chances for EET. This seems actually to be the case, considering the results shown in Section S8; in fact, the full-QM approach shows a larger number of transitions between *S*_2_*S*_0_ and *S*_0_*S*_2_ excitons with respect to the EC and TC schemes in the first 300 fs, while the situation is reversed at later times.

To test the approximation used in the calculation of the analytical gradients of the energy (see Section 2), we compared them with the numerical gradients, evaluated at selected geometries, as shown in detail in the Supporting Information, Section S9. In general, the agreement of approximated analytical gradients with the numerical ones is quite good for the EC approach; in fact, in that case, large relative errors are found only for small values of the gradient, where the numerical evaluation is less accurate. A less regular behavior, and slightly worse agreement with the numerical gradients, is shown by the TC approximate gradients.

## 4 Conclusions

We report the formulation and implementation of a method for surface hopping dynamics in the framework of the Frenkel exciton model, with energies and couplings evaluated with a semiempirical QM/MM approach. The full treatment of nonadiabatic dynamics and photoreactivity within each chromophore is complemented by excitation transfer between chromophores. The Coulomb exciton couplings are either computed directly from the exact expression (the EC approach) or approximated resorting to transition atomic charges (TC approach). Notice that the approximation of neglecting the exchange exciton interaction is inherent in the semiempirical NDO approach. The present methodology was tested in the study of the photoisomerization dynamics of 2S-TTABP, for which both weakly (nπ*) and strongly (ππ*) coupled excitons were considered. Overall, the two exciton approaches (EC and TC) showed very close matching results in terms of absorption spectra, lifetimes, and photoisomerization quantum yields. The results obtained with the exciton model are also in good agreement with the full-QM ones. The largest discrepancy is found in the lifetimes after ππ* excitation, about four times smaller at the full-QM level. This is most likely due to differences in the PESs of the ππ* states. The photoisomerization quantum yields obtained are also in good agreement with the experimental results.

According to the present results, the computationally cheaper TC approach can be safely employed, even when the distance between the chromophores is small. We emphasize that 2S-TTABP should be considered quite a hard test for the exciton model because the two chromophores are close in space and do interact “through-bond”. Moreover, the singlet combinations of two low-lying triplets may have non-negligible interactions with the localized singlets, affecting the nonadiabatic dynamics. We can conclude that sparser assemblies of chromophores would certainly be treated more accurately by the present approach.

## Supporting Information Available

Expression of *E*_*gs*_ in terms of QM, MM, and QM/MM contributions; reference states used in the diabatization calculations; exciton couplings in 2S-TTABP; kinetic model used; adiabatic populations; results obtained with the exciton EC model; comparison between numerical and analytical gradients; and Cartesian coordinates of selected molecular geometries (PDF)
