# Supplementary material for: Surface Hopping Dynamics with the Frenkel Exciton Model in a Semiempirical Framework
Source: J Chem Theory Comput. 2021 Nov 29;17(12):7373–83. doi: 10.1021/acs.jctc.1c00942 (PMC8675141; doi:10.1021/acs.jctc.1c00942)
Supplement: Supplementary file 1 — ct1c00942_si_001.pdf [file ct1c00942_si_001.pdf]

Support Information for:

"Surface hopping dynamics with Frenkel exciton model in semiempirical framework"

Eduarda Sangiogo Gil, Giovanni Granucci, and Maurizio Persico

Dipartimento di Chimica e Chimica Industriale, University of Pisa, via Moruzzi 13, 56124 Pisa, Italy

Contents

S1 Energy of the ground state **3**

S2 Reference states considered **4**

S3 Exciton coupling **5**

S4 Thermalizations and sampling **7**

S5 Kinetic model **9**

S6 Adiabatic Populations **10**

S7 Results for 2S-TTABP obtained at exciton EC level **11**

S8 Diabatic transitions analysis **15**

S9 Gradients checking **17**

S10 Geometries **21**

S1 Energy of the ground state

We want to assess here about the validity of the expression we have used for the ground state energy E_{gs} (equation 4 of the main text, repeated here for readers' convenience)

$$E_{gs} = \sum_{a}^{N_C} \left(E_0^{(a)} - E_{MM} \right) + E_{MM}$$

The analysis reported in the main text, based on the three terms of the electronic Hamiltonian (QM, MM and QM/MM), is limited for simplicity to the case of a system consisting of two chromophores. Here we extend the analysis to the general case of a system having $N_C + 1$ components (the N_C chromophores and the environment). The ground state energy of chromophore a, namely $E_0^{(a)}$, is defined as

$$\begin{aligned} E_0^{(a)} =& E(QM,a) + \sum_{b(\neq a)} E(MM,b) + E(MM,env) + \sum_{b(\neq a)} E(QM/MM,a,b) \\ &+ E(QM/MM,a,env) + \sum_{b(\neq a)} E(MM,b,env) + \tfrac{1}{2} \sum_{b\neq c(\neq a)} E(MM,b,c) \end{aligned}$$

where env labels the environment, $E(QM,a)$ is the QM energy of the a chromophore, $E(MM,b)$ is the MM energy of the b chromophore, $E(MM,b,env)$ is the MM interaction energy between b and the environment, and $E(QM/MM,a,b)$ is the QM/MM interaction energy between the QM chromophore a and the MM chromophore b. The total MM energy is

$$E_{MM} = \sum_b E(MM,b) + E(MM,env) + \sum_b E(MM,b,env) + \tfrac{1}{2} \sum_{b\neq c} E(MM,b,c).$$

We obtain therefore the following expression for E_{gs}

$$\begin{aligned} E_{gs} =& \sum_a E(QM,a) + (N_C - 1) \sum_a E(MM,a) + N_C\ E(MM,env) + \sum_{a\neq b} E(QM/MM,a,b) \\ &+ \sum_a E(QM/MM,a,env) + (N_C - 1) \sum_a E(MM,a,env) + \tfrac{1}{2} N_C \sum_{a\neq b} E(MM,a,b) \\ &- \sum_{a\neq b} E(MM,a,b) - (N_C - 1) \sum_a E(MM,a) - (N_C - 1) E(MM,env) \\ &- (N_C - 1) \sum_a E(MM,a,env) - \tfrac{1}{2}(N_C - 1) \sum_{a\neq b} E(MM,a,b) \end{aligned}$$

which simplifies to

$$E_{gs} = \sum_a E(QM,a) + E(MM,env) + \sum_a E(QM/MM,a,env) + \sum_{a\neq b} E(QM/MM,a,b) - \tfrac{1}{2} \sum_{a\neq b} E(MM,a,b)$$

where the last term is a way to compensate for double counting of the interaction between chromophores.

S2 Reference states considered

Figure S1 presents a schematic representation of the 13 model states used as references for the diabatization procedure in the full-QM calculations. For the construction of the diabatic states it was considered that each monomer (a and b) has an active space of 6 electrons in 4 orbitals (the total active space is 12 electrons in 8 orbitals).

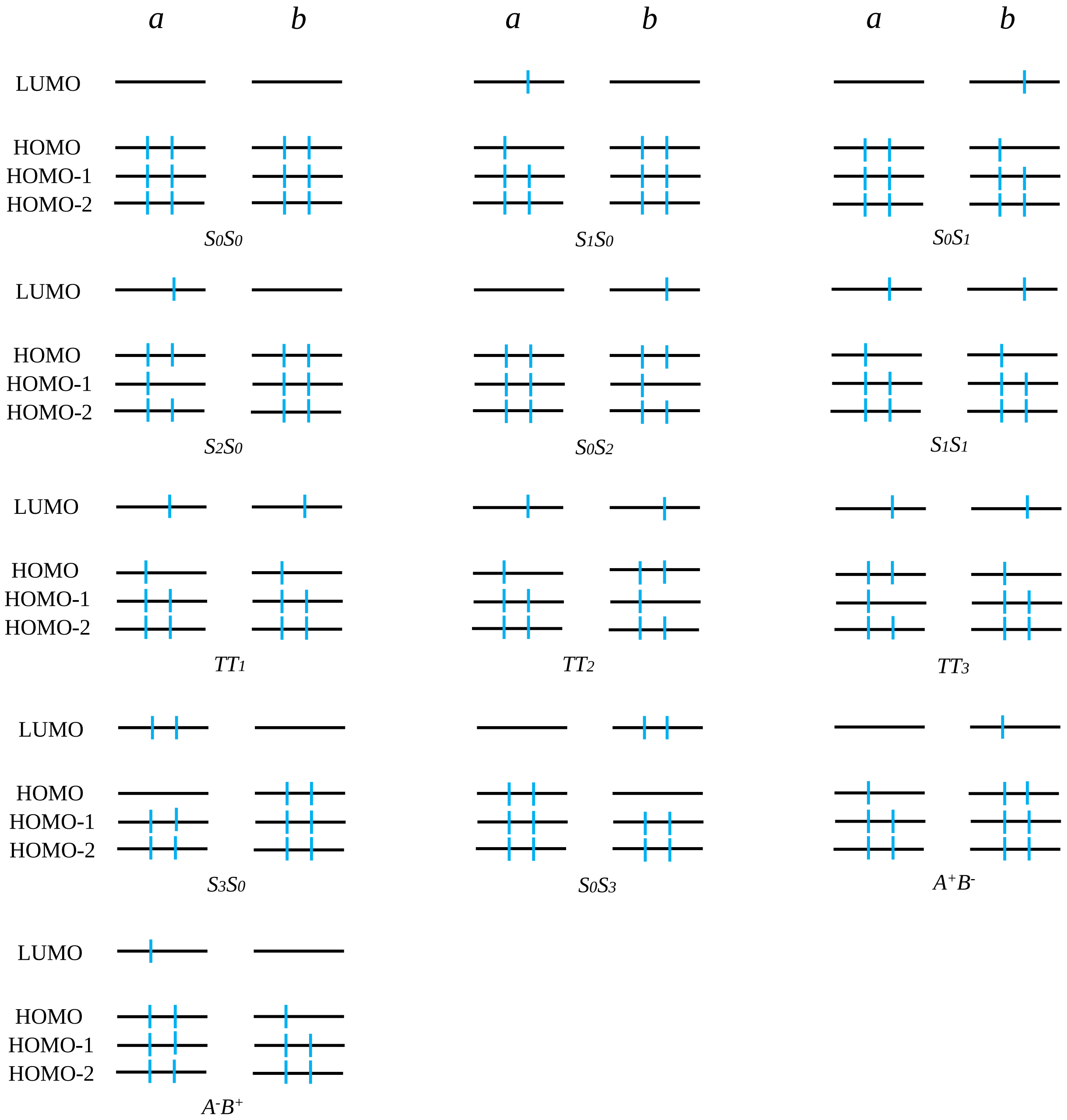

Figure S1: Schematic representation of the model states used for the construction of the diabatic states in the full-QM approach.

S3 Exciton coupling

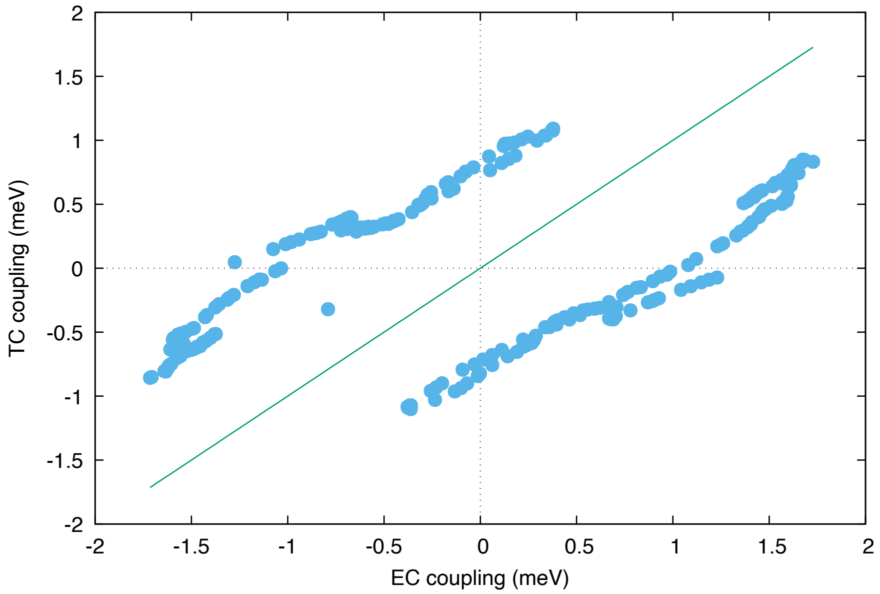

Figure S2: Electronic couplings between $n\pi^*$ excitons, evaluated at geometries randomly sampled from a ground state thermalization trajectory. Blue circles: TC couplings (y axis) versus EC couplings (x axis), in meV. The green line is added as a guide for the eye.

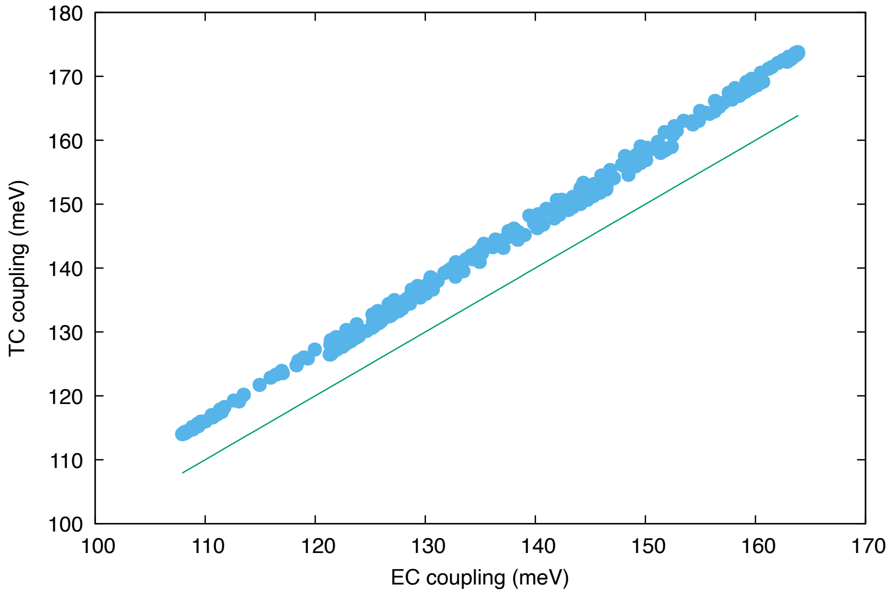

Figure S3: Electronic couplings between $\pi\pi^*$ excitons, evaluated at geometries randomly sampled from a ground state thermalization trajectory. Blue circles: TC couplings (y axis) versus EC couplings (x axis), in meV. The green line (bisector of the quadrant) is added as a guide for the eye.

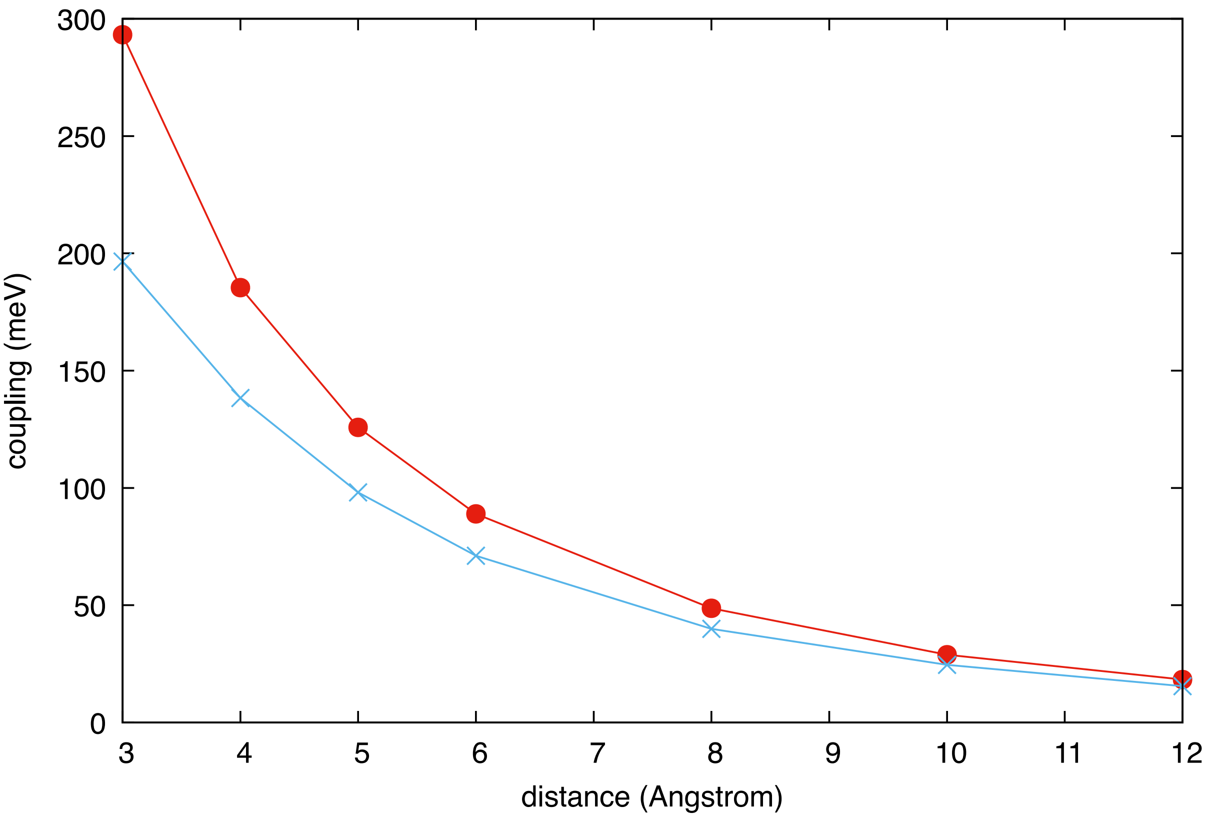

Figure S4: Excitonic $\pi\pi^*/\pi\pi^*$ couplings for two stacked *trans*-azobenzene molecules, with respect to the stacking distance. The two stacked molecules are superimposed (i.e., there is no slip). Blue crosses: EC couplings. Red circles: TC couplings.

S4 Thermalizations and sampling

Table S1: Fraction of trajectories starting on each adiabatic state. The exciton approaches TC and EC share the same set of starting conditions.

$n\pi^*$ excitation					
method	$S_1^{(d)}$	$S_2^{(d)}$			
full-QM	0.48	0.52			
exciton	0.51	0.49			
$\pi\pi^*$ excitation					
method	$S_3^{(d)}$	$S_4^{(d)}$	$S_5^{(d)}$	$S_6^{(d)}$	$S_7^{(d)}$
full-QM		0.09	0.61	0.29	0.01
exciton	0.08	0.71	0.16	0.05	

Table S2: Initial population of each diabatic state. The exciton approaches TC and EC share the same set of starting conditions.

$n\pi^*$ excitation									
method	S_1S_0	S_0S_1							
full-QM	0.53	0.47							
exciton	0.52	0.48							
$\pi\pi^*$ excitation									
method	S_1S_0	S_0S_1	S_2S_0	S_0S_2	S_0S_3	S_3S_0	TT_1	TT_2	TT_3
full-QM	0.03		0.48	0.46				0.01	0.02
exciton			0.44	0.40	0.08	0.08			

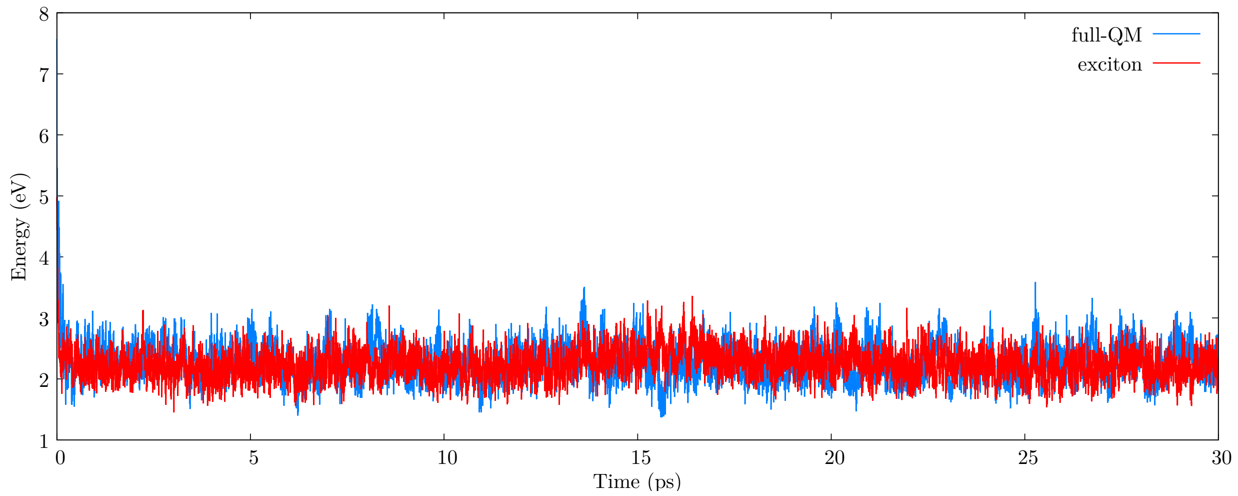

Figure S5: Time evolution of the ground state energy during the thermalization. Full-QM approach (blue line) and exciton model (red line). The energies are relative to that of the ground state minimum.

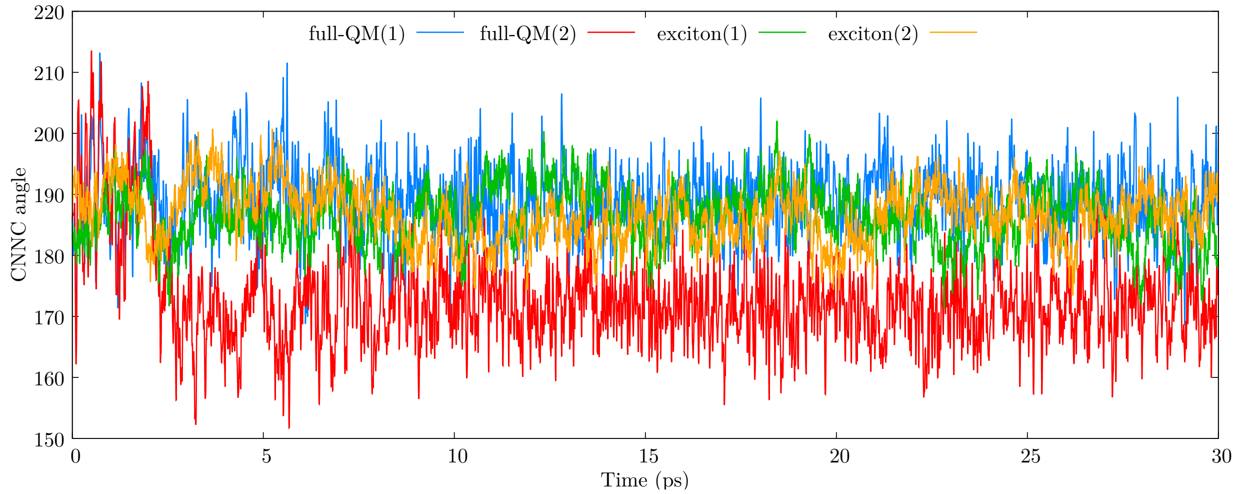

Figure S6: Time evolution of the CNNC dihedrals during the full-QM and exciton model thermalizations.

S5 Kinetic model

$P_{n\pi^*}$ is defined as the sum of S_1S_0 and S_0S_1 populations, and $P_{\pi\pi^*}$ the sum of the S_nS_0 and S_0S_n populations with $n > 1$. In the case of $n\pi^*$ excitation, we fitted $P_{n\pi^*}$ with a delayed exponential function:

$$P_{n\pi^*}(t) = \begin{cases} 1 & \text{for} \quad t < t_0 \\ e^{-(t-t_0)/\tilde{\tau}_1} & \text{for} \quad t > t_0 \end{cases} \tag{1}$$

where t_0 is a delay time. The overall lifetime of $n\pi^*$ states was defined as $\tau_1 = \tilde{\tau}_1 + t_0$.

For the simulations after $\pi\pi^*$ excitation we considered a two-step irreversible first-order decay kinetics. In the first step the $\pi\pi^*$ states decay exponentially to the $n\pi^*$ ones and, in the full-QM treatment, to the long lived state TT_1 as well. The respective rate constant are k_2 and k_2'. Then:

$$P_{\pi\pi^*}(t) = e^{-(k_2+k_2')t} \tag{2}$$

The lifetime of the $\pi\pi^*$ states is $\tau_2 = (k_2 + k_2')^{-1}$ (here and in the following the k_2' constant must be omitted, or set to zero, when dealing with the TC and EC schemes, which do not take into account the TT_n states). The $n\pi^*$ states are populated at a rate $k_2 e^{-(k_2+k_2')t}$ and decay according to a law $f(t)$ similar to equation 1, where we shall replace $\tilde{\tau}_1$ with k_1^{-1} (notice that the parameters t_0 and $\tilde{\tau}_1$ are determined independently for $n\pi^*$ and $\pi\pi^*$ excitation). $P_{n\pi^*}$ can be represented as the convolution of the population rate with the decay law:

$$P_{n\pi^*}(t) = \int_0^t k_2 \; P_{\pi\pi^*}(t') \; f(t-t') \; dt' \tag{3}$$

Then, for $t \leq t_0$ we get

$$P_{n\pi^*}(t) = R \; \left[1 - e^{-t/\tau_2}\right] \tag{4}$$

and, for $t > t_0$

$$\begin{aligned} P_{n\pi^*}(t) &= \frac{k_2}{k_2 + k_2' - k_1} \left[e^{-k_1(t-t_0)} - e^{-(k_2+k_2')(t-t_0)}\right] + \frac{k_2}{k_2 + k_2'} \left[e^{-(k_2+k_2')(t-t_0)} - e^{-(k_2+k_2')t}\right] \\ &= R\frac{\tilde{\tau}_1}{\tilde{\tau}_1 - \tau_2} \left[e^{-(t-t_0)/\tilde{\tau}_1} - e^{-(t-t_0)/\tau_2}\right] + R\left[e^{-(t-t_0)/\tau_2} - e^{-t/\tau_2}\right] \end{aligned} \tag{5}$$

where R is the branching ratio for the decay of the $\pi\pi^*$ states to the $n\pi^*$ ones: $R = k_2/(k_2 + k_2')$. When fitting the full-QM results, R is set equal to $1 - P_{TT_1}(\infty)$, where $P_{TT_1}(\infty) = 0.12$ is the average population of the TT_1 state at long times ($t > 1$ ps). For the TC and EC schemes, $R = 1$.

S6 Adiabatic Populations

In Figures S7 and S8 we present the fraction of trajectories running on a given adiabatic state at a given time, after $n\pi^*$ and $\pi\pi^*$ excitation, respectively.

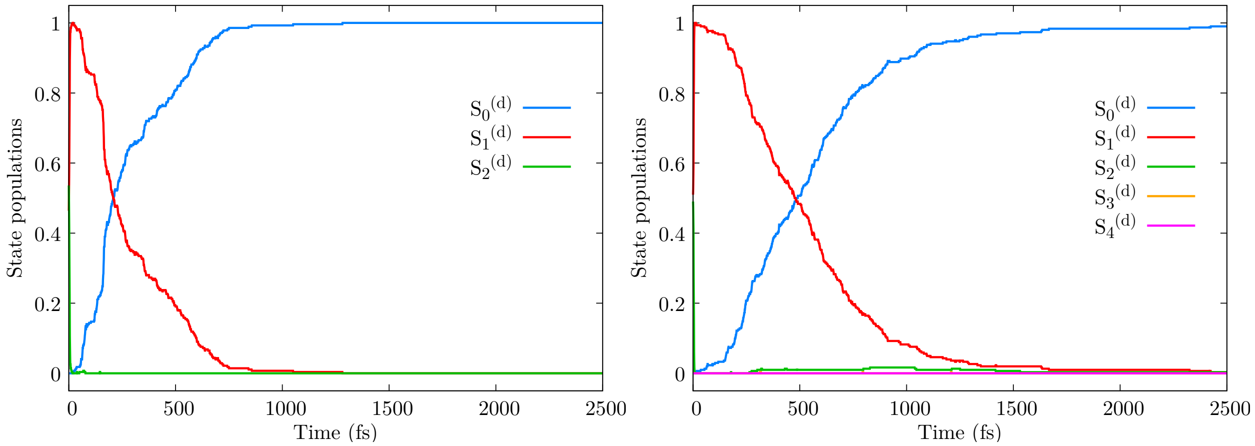

Figure S7: Time evolution of the adiabatic states populations after $n\pi^*$ excitation. Left panel: full-QM approach. Right panel: exciton TC scheme. The corresponding results for the exciton EC scheme, very similar to the TC ones, are shown in the compilation of the EC results, section S7.

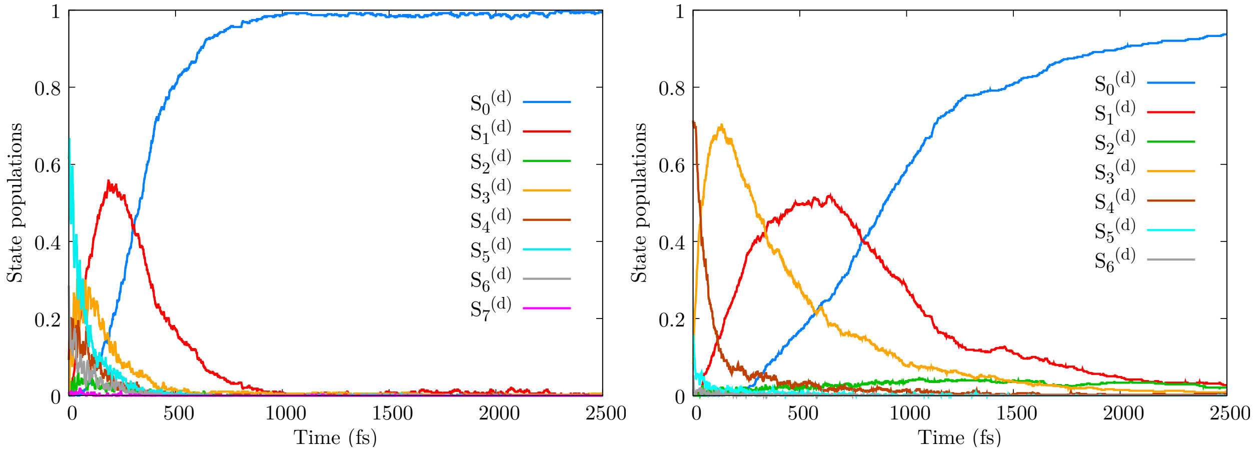

Figure S8: Time evolution of the adiabatic states populations after $\pi\pi^*$ excitation. Left panel: full-QM approach. Right panel: exciton TC scheme. The corresponding results for the exciton EC scheme, very similar to the TC ones, are shown in the compilation of the EC results, section S7.

S7 Results for 2S-TTABP obtained at exciton EC level

In order to avoid redundancy, in some cases we decided to report in the main text only the result obtained with the exciton TC approach, omitting the almost identical computed at the exciton EC level, which are instead presented here.

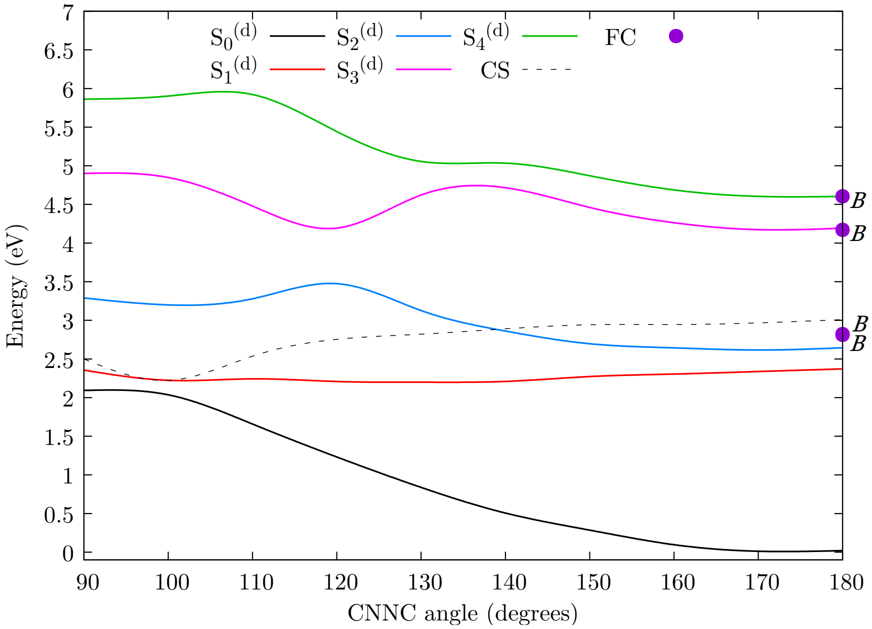

Figure S9: Exciton EC approach: potential energy curves of the lowest singlet states as function as one CNNC dihedral. All the other coordinates have been optimized for each state (except for the last two states shown, which were computed at the $S_0^{(d)}$ optimal geometries). Franck Condon (FC) points are represented as dots, with symmetry labels (C_2 symmetry). The dashed curve represents the $S_0^{(d)}/S_1^{(d)}$ crossing seam (CS).

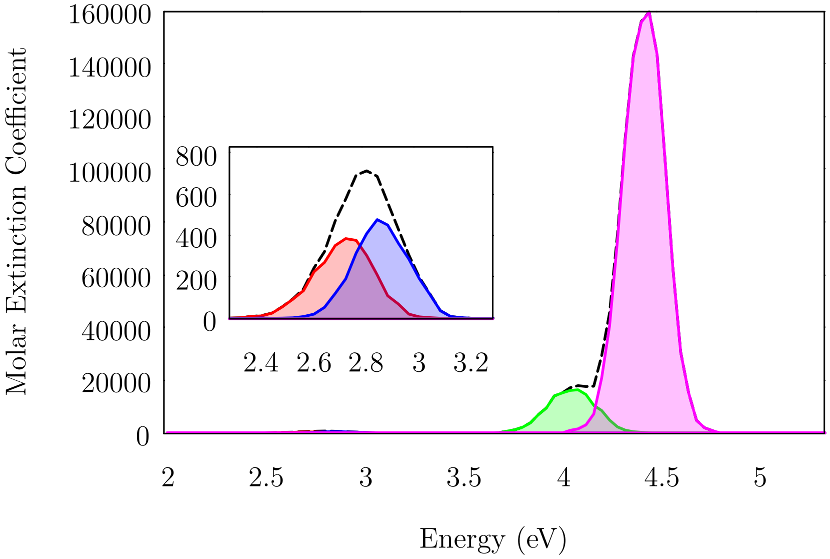

Figure S10: Simulated absorption spectrum at the exciton EC level. The contribution of each adiabatic state is shown. The dashed line represents the total spectrum.

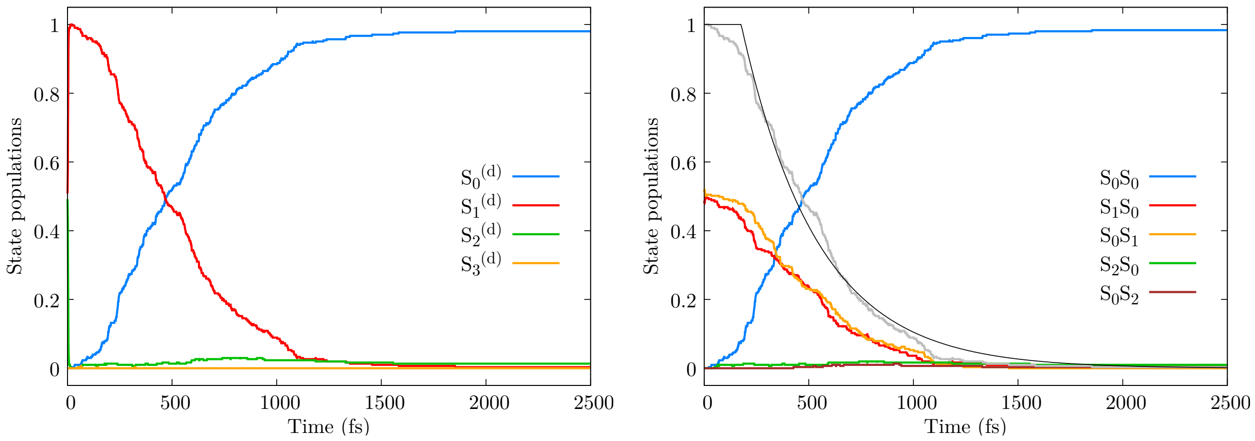

Figure S11: State populations as functions of time for the trajectories starting on $n\pi^*$ states. Adiabatic states on the left and excitonic states on the right.

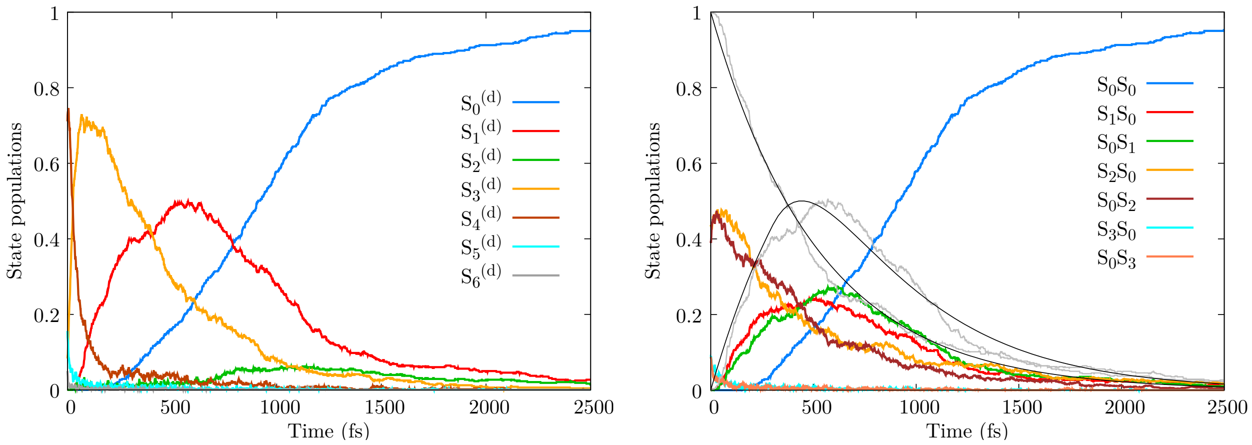

Figure S12: State populations as functions of time for the trajectories starting on $\pi\pi^*$ states. Adiabatic states on the left and excitonic states on the right.

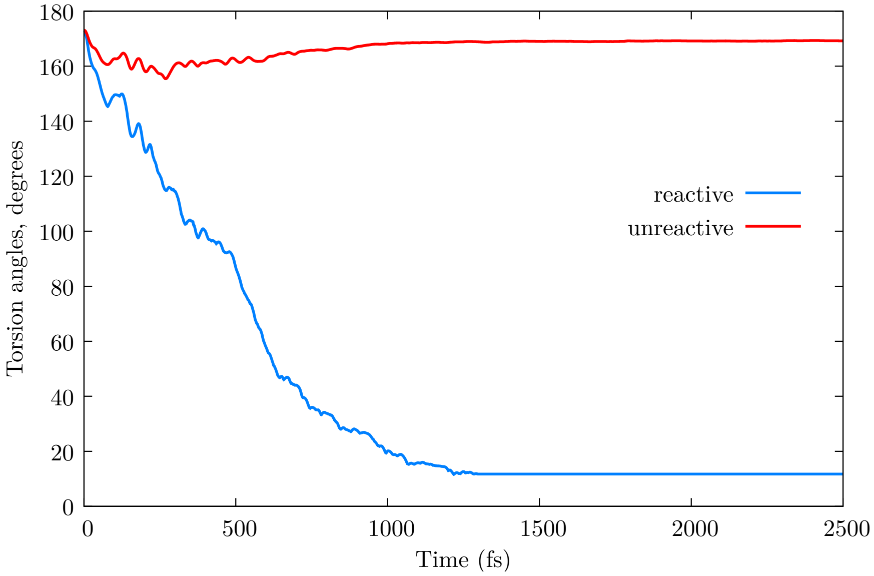

Figure S13: CNNC torsion angle as a function of time at the exciton EC level, averaged separately for reactive and unreactive trajectories, after $n\pi^*$ excitation. Only the CNNC dihedral of the isomerizing monomer was taken into account when averaging over the reactive trajectories, whereas both CNNC dihedrals were averaged for the unreactive trajectories.

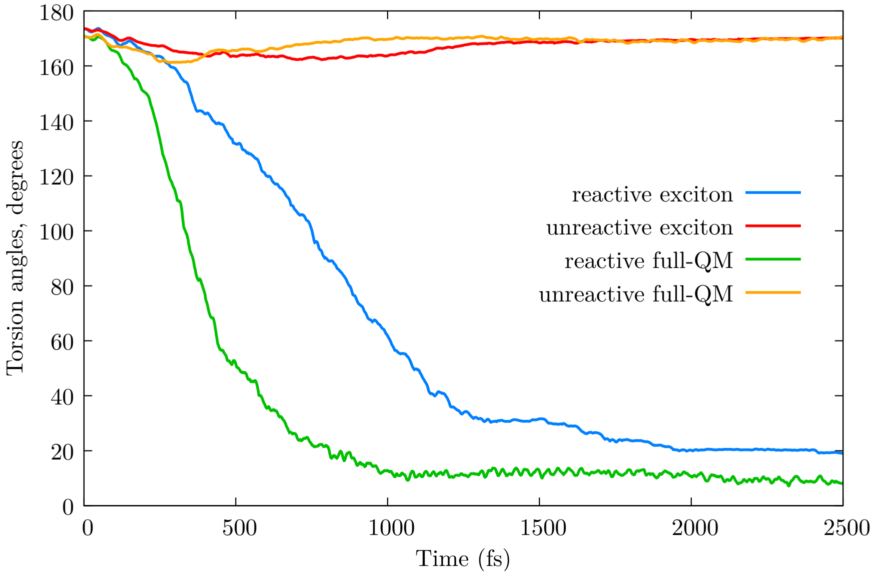

Figure S14: CNNC torsion angle as a function of time at the full-QM and exciton TC levels, averaged separately for reactive and unreactive trajectories, after $\pi\pi^*$ excitation. Only the CNNC dihedral of the isomerizing monomer was taken into account when averaging over the reactive trajectories, whereas both CNNC dihedrals were averaged for the unreactive trajectories.

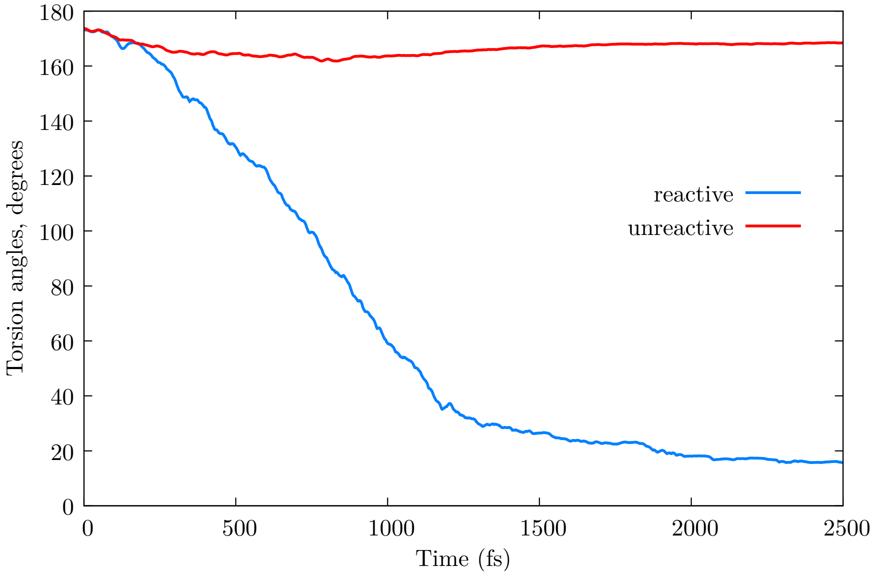

Figure S15: CNNC torsion angle as a function of time at the exciton EC level, averaged separately for reactive and unreactive trajectories, after $\pi\pi^*$ excitation. Only the CNNC dihedral of the isomerizing monomer was taken into account when averaging over the reactive trajectories, whereas both CNNC dihedrals were averaged for the unreactive trajectories.

S8 Diabatic transitions analysis

We report in the present section the fraction $T(i \longrightarrow f)$ of transitions per trajectory from the diabatic state i to f, i.e.

$$T(i \longrightarrow f) = \frac{\text{\# of transitions from } i \text{ to } f}{\text{total \# of trajectories}}$$

at selected time intervals. Only the dinamics simulations after $\pi\pi^*$ excitation are considered. Only transitions involving at least one of the S_0S_n and/or S_nS_0, with $n \geq 1$ are considered. Moreover, the transitions to the diabatic ground state S_0S_0 are not shown.

Table S3: Full-QM $\pi\pi^*$ dynamics, diabatic transitions per trajectory in the time intervals 0-300 fs and 300-600 fs.

		0-300 fs			300-600 fs		
i_{dia}	f_{dia}	$T(i \longrightarrow f)$	$T(f \longrightarrow i)$	$T(net)$	$T(i \longrightarrow f)$	$T(f \longrightarrow i)$	$T(net)$
S_1S_0	S_0S_2	0.48	0.49	-0.010	0.04	0.05	-0.003
S_0S_1	S_2S_0	0.19	0.23	-0.039	0.01	0.02	-0.010
S_2S_0	S_0S_2	6.03	6.06	-0.031	0.29	0.27	0.026
S_1S_0	S_0S_1	0.47	0.43	0.031	0.06	0.07	-0.010
S_1S_0	S_2S_0	1.59	1.97	-0.381	0.22	0.23	-0.008
S_0S_1	S_0S_2	1.16	1.49	-0.329	0.17	0.20	-0.026
S_1S_0	S_3S_0	0.04	0.03	0.008	0.04	0.03	0.010
S_0S_1	S_0S_3	0.06	0.05	0.005	0.03	0.05	0.026
S_2S_0	TT_1	0.59	0.57	0.013	0.05	0.05	0.000
S_0S_2	TT_1	0.72	0.68	0.033	0.03	0.05	-0.021
S_1S_0	TT_1	0.14	0.16	-0.015	0.00	0.01	-0.010
S_0S_1	TT_1	0.10	0.08	0.010	0.00	0.01	-0.010
S_2S_0	TT_2	0.69	0.69	-0.005	0.06	0.09	-0.005
S_2S_0	TT_3	0.29	0.26	0.018	0.02	0.02	0.008
S_0S_2	TT_2	0.28	0.24	0.031	0.00	0.00	0.000
S_0S_2	TT_3	0.63	0.42	-0.008	0.04	0.03	0.008

Table S4: Exciton TC $\pi\pi^*$ dynamics, diabatic transitions per trajectory in the time intervals 0-300 fs and 300-600 fs.

		0-300 fs			300-600 fs		
i_{dia}	f_{dia}	$T(i \longrightarrow f)$	$T(f \longrightarrow i)$	$T(net)$	$T(i \longrightarrow f)$	$T(f \longrightarrow i)$	$T(net)$
S_1S_0	S_0S_2	0.00	0.01	-0.008	0.00	0.01	-0.008
S_0S_1	S_2S_0	0.00	0.01	-0.008	0.00	0.00	0.000
S_2S_0	S_0S_2	4.56	4.56	-0.004	1.15	1.14	0.008
S_1S_0	S_0S_1	0.01	0.01	0.004	0.01	0.01	0.002
S_1S_0	S_2S_0	0.06	0.27	-0.222	0.05	0.19	-0.142
S_0S_1	S_0S_2	0.05	0.25	-0.202	0.06	0.19	-0.127

Table S5: Exciton EC $\pi\pi^*$ dynamics, diabatic transitions per trajectory in the time intervals 0-300 fs and 300-600 fs.

		0-300 fs			300-600 fs		
i_{dia}	f_{dia}	$T(i \longrightarrow f)$	$T(f \longrightarrow i)$	$T(net)$	$T(i \longrightarrow f)$	$T(f \longrightarrow i)$	$T(net)$
S_1S_0	S_0S_2	0.00	0.01	-0.010	0.00	0.00	-0.004
S_0S_1	S_2S_0	0.00	0.01	-0.010	0.00	0.00	-0.002
S_2S_0	S_0S_2	3.94	3.84	0.106	1.15	1.12	-0.004
S_1S_0	S_0S_1	0.01	0.01	0.002	0.00	0.00	-0.002
S_1S_0	S_2S_0	0.06	0.29	-0.226	0.06	0.17	-0.108
S_0S_1	S_0S_2	0.05	0.25	-0.197	0.06	0.21	-0.147

S9 Gradients checking

We have assumed approximations in the evaluation of the gradients for both exciton approaches. For the exciton with TC approximation, it was considered that the derivative TrESP remain constant in the gradient calculation. Similarly, the transition densities remain constant in the gradient calculation for the exciton when the Coulomb integral was calculated exactly. In order to check the accuracy of the approximated analytical gradients, we compared them with the numerical gradients, evaluated using a two point finite difference formula, with a step size of 0.01 Å. The comparison is performed at selected geometries and for selected states: *i*) state $S_4^{(d)}$ (corresponding to a $\pi\pi^*$ excitation) close to the ground state equilibrium geometry; *ii*) state $S_4^{(d)}$ close to the conical intersection with $S_3^{(d)}$ (a $\pi\pi^*/n\pi^*$) conical intersection); *iii*) state $S_2^{(d)}$ (which corresponds to a $n\pi^*$ excitation) far from the conical intersection with the ground state and *iv)* state $S_2^{(d)}$ state close to the conical intersection with the ground state.

Table S6: Root mean square error (in Kcal/Å) of the approximate analytical gradients (evaluated either with the EC or the TC approach) with respect to the numerical ones.

	Geometries			
exciton	*i*	*ii*	*iii*	*iv*
EC	0.3899	0.4096	0.4005	0.4011
TC	1.3090	0.6764	0.3981	0.8636

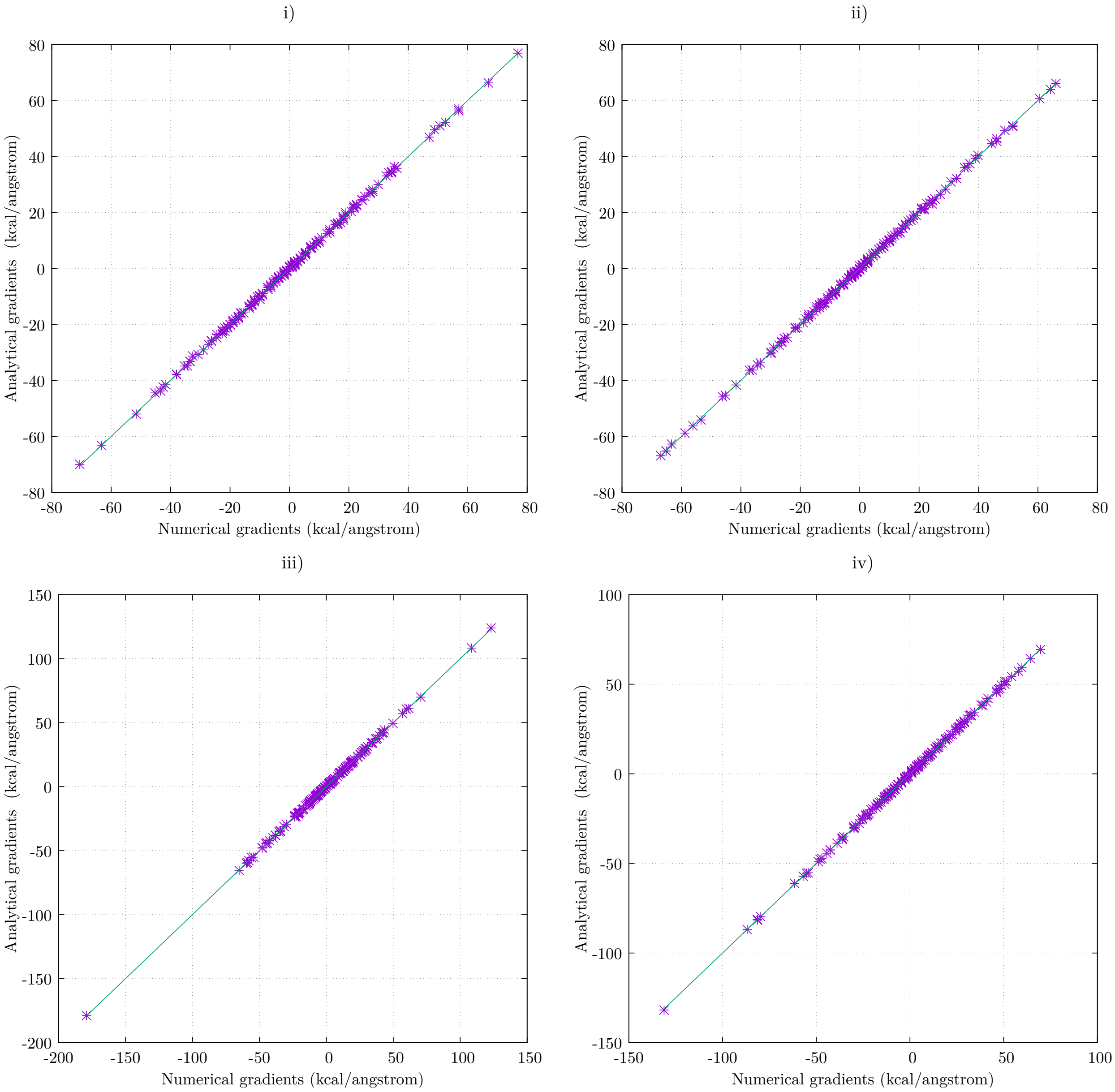

Figure S16: Numerical gradients *vs.* the analytical EC ones.

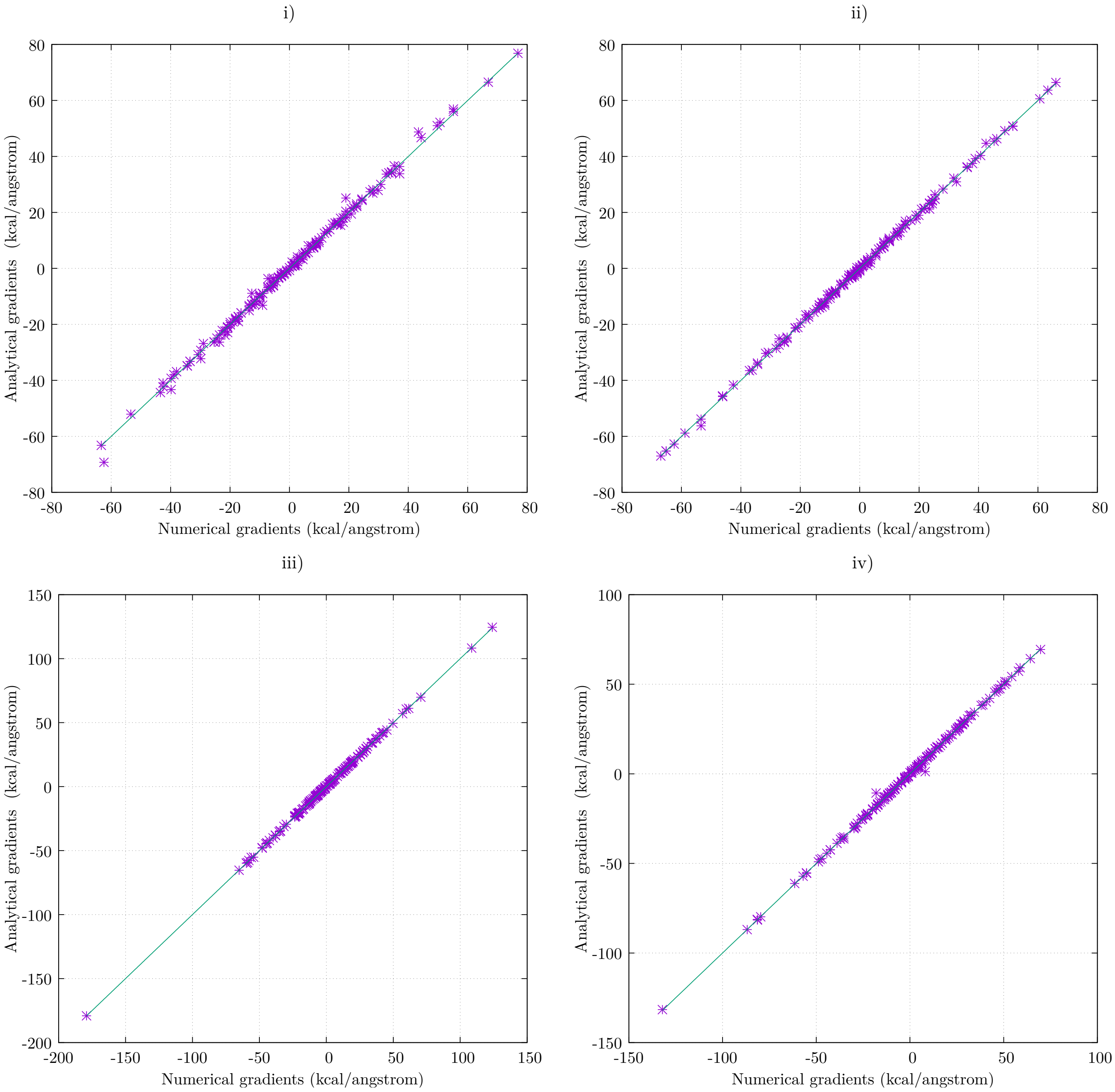

Figure S17: Numerical gradients *vs.* the analytical TC ones.

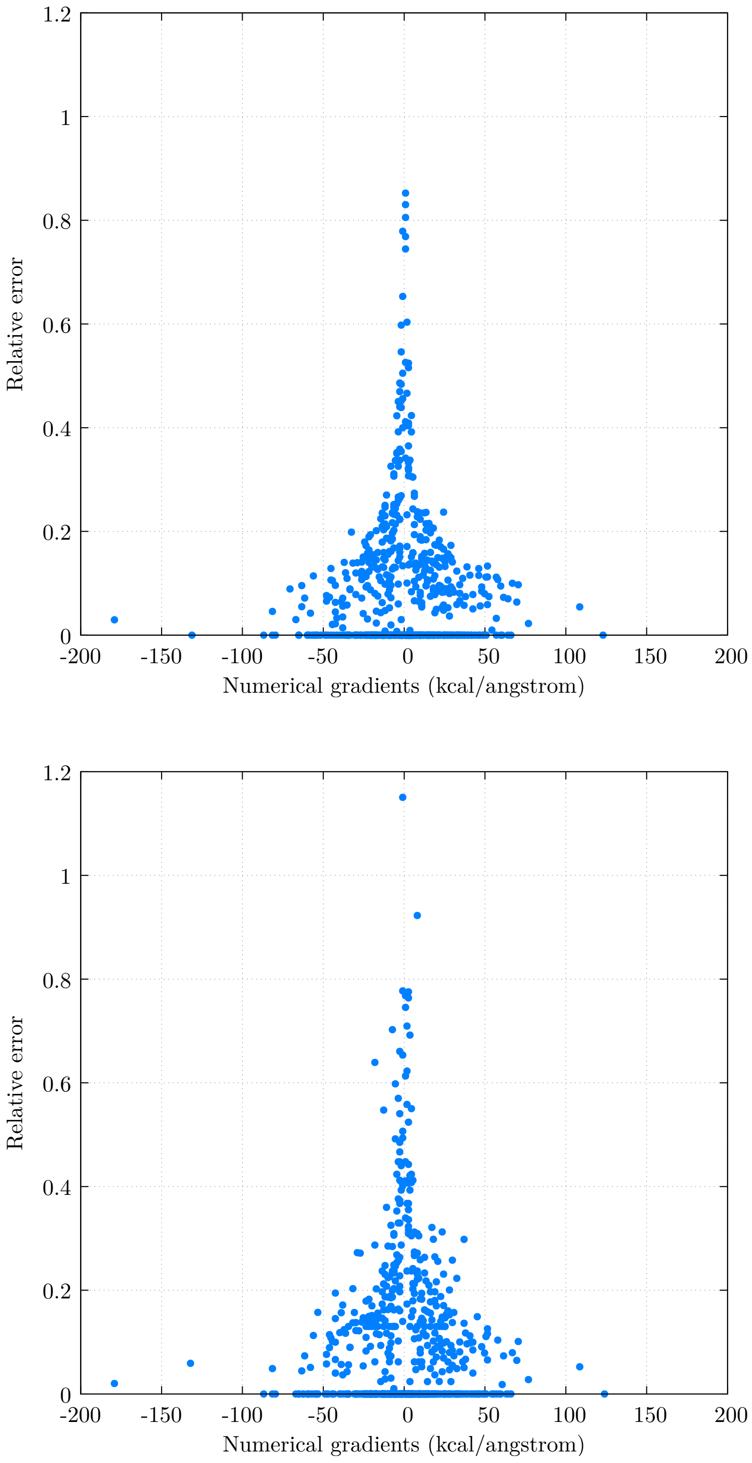

Figure S18: Top panel: absolute value of the relative error of the approximate analytical EC gradients with respect to the numerical ones. Bottom panel: the same for the TC gradients.

S10 Geometries

We report here the coordinates (in Angstrom) of some important conformations.

- Ground state *trans-trans* minimum at full-QM level

```
C -0.00669321 -0.02272672 0.08473552
C 1.45598076 -0.02272672 0.08473552
C 2.17203944 1.14700477 0.08473552
C 3.52393848 1.16165630 -0.00493897
H 4.06056729 2.13377242 -0.03693142
C 4.23224002 -0.02041109 -0.09043316
C 3.52113165 -1.20811655 -0.00572666
C 2.17124462 -1.19272947 0.07098339
H 1.62585159 -2.16013537 0.11742573
H 4.06163689 -2.17958530 -0.01999437
N 5.64861580 -0.15928764 -0.31450220
N 6.26898751 0.84937380 -0.78662400
C 7.64782217 0.66253842 -1.16097207
C 8.15509789 1.58875434 -2.05981425
C 9.39520243 1.43241977 -2.57563740
H 9.76913578 2.17883427 -3.30948444
C 10.20079807 0.37818842 -2.22892944
C 9.72230076 -0.48646397 -1.27798704
C 8.47929867 -0.36142890 -0.75345764
H 8.12096266 -1.10574204 -0.01070843
H 10.36090217 -1.33674118 -0.95466031
C 11.50629182 0.17637161 -2.85654657
H 7.53215683 2.45462292 -2.37329071
H 1.62389464 2.11311675 0.12227406
H -0.46723030 -1.00192962 0.36783259
H -0.46973750 0.76390563 0.73363950
S -0.63361185 0.42677404 -1.52624044
C -0.74026136 -0.77469296 -2.84576138
H -1.07075380 -1.74562667 -2.40030430
H -1.59365181 -0.37731925 -3.45165401
C 0.53477872 -0.83952925 -3.55934343
C 1.40654871 -1.88128950 -3.37361219
```

C 2.62855219 -1.90695158 -3.95349740

H 3.30462951 -2.76749488 -3.76194799

H 1.10733610 -2.72528756 -2.71531468

C 0.92073708 0.15330628 -4.42377577

H 0.22691727 0.99950967 -4.61640223

C 2.14067696 0.15737197 -5.01231870

H 2.42704603 0.99936507 -5.67837427

C 3.04598376 -0.86161811 -4.75603905

N 4.37930116 -0.66953341 -5.26298634

N 5.20307174 -1.63744839 -5.15861969

C 6.58483936 -1.38264252 -5.47130443

C 7.15711822 -0.13724066 -5.65142348

C 8.50022101 -0.00648284 -5.74561798

H 8.93239270 1.01015300 -5.86791836

H 6.51649432 0.76922900 -5.69920279

C 7.42416131 -2.48674246 -5.47998418

H 6.99898375 -3.50724097 -5.36868459

C 8.76520680 -2.32649884 -5.58391887

H 9.41917518 -3.22417903 -5.55234252

C 9.34292258 -1.08597469 -5.67861349

C 10.79611513 -0.92022660 -5.68134517

H 11.14663341 0.07960768 -6.03904485

H 11.35251502 -1.69743584 -6.26424345

S 11.43806152 -1.16770792 -4.03134691

H 12.30665777 -0.17702163 -2.15742048

H 11.90379143 1.07485738 -3.39120057

- Minimum of the $S_0^{(d)}/S_1^{(d)}$ conical intersection at full-QM level

C -0.00108779 -0.01129816 0.07735354

C 1.45989113 -0.01129816 0.07735354

C 2.17240728 1.16296880 0.07735354

C 3.52507594 1.17909036 0.03243767

H 4.06567853 2.14813539 0.02003246

C 4.23852306 -0.01297895 -0.00227727

C 3.52332923 -1.20510261 -0.00750147

C 2.17312512 -1.18194481 0.04595987

H 1.62461042 -2.14851085 0.03926611

H 4.05598878 -2.17658307 -0.05734221

N 5.62963763 0.02342387 0.05861487

N 6.61847029 -0.73319699 0.26826858

C 7.50637556 -1.36066030 -0.63646954

C 7.40690381 -1.27689630 -2.01589619

C 8.34686124 -1.84375138 -2.80360443

H 8.24361447 -1.75702017 -3.90719695

C 9.41736435 -2.53166386 -2.28946221

C 9.51807927 -2.59933774 -0.92378070

C 8.59225023 -2.04233474 -0.10658970

H 8.69263695 -2.15175414 0.99508864

H 10.37010473 -3.15268209 -0.47340393

C 10.39416181 -3.18058021 -3.16230362

H 6.55368574 -0.73780173 -2.47938380

H 1.62433543 2.12822705 0.09341779

H -0.46307732 -0.95131863 0.46844943

H -0.47645457 0.84274369 0.62048012

S -0.55708278 0.20603045 -1.61169303

C -1.33421443 -1.15993468 -2.46460085

H -1.89142786 -1.77007820 -1.71253368

H -2.09097962 -0.65923733 -3.11945972

C -0.27955165 -1.87866548 -3.17609279

C 0.26091789 -3.03669063 -2.66798226

C 1.33233993 -3.63195138 -3.23698570

H 1.75546355 -4.54849802 -2.77572237

H -0.17215879 -3.47812740 -1.74585275

C 0.24146587 -1.39558020 -4.35337543

H -0.21262033 -0.49707014 -4.81970203

C 1.31773387 -1.96825377 -4.94073993

H 1.73015846 -1.53215587 -5.87437674

C 1.92292497 -3.07674508 -4.36048564

N 3.18945291 -3.46684509 -4.92437754

N 3.76587827 -4.49428100 -4.43146765

C 5.12878698 -4.74767961 -4.82380995

- Ground state *trans*-*trans* minimum at exciton TC level

```
C 0.000000 0.000000 0.000000
C 1.143584 0.000000 0.000000
C 1.806746 1.194284 0.000000
C 3.154906 1.268521 -0.092579
H 3.645257 2.265177 -0.123552
H 1.213614 2.133383 0.039799
C 1.904670 -1.143500 0.014483
H 1.424320 -2.143873 0.073057
C 3.252777 -1.095984 -0.078348
H 3.835142 -2.043105 -0.099724
C 3.915007 0.119168 -0.183145
N 5.320911 0.040595 -0.456208
N 5.959712 1.137566 -0.612179
C 7.333102 1.051754 -1.018474
C 8.060665 -0.104477 -1.228435
C 9.321117 -0.049981 -1.715768
H 9.858310 -1.005929 -1.897097
H 7.604022 -1.094789 -1.014408
C 7.963385 2.260016 -1.273887
H 7.419709 3.213268 -1.095946
C 9.223728 2.288447 -1.765101
H 9.686406 3.273940 -1.989471
C 9.922802 1.141521 -2.030174
C 10.975267 1.185972 -2.489448
C -0.660228 0.598992 -2.512091
C 0.372144 0.677051 -3.009383
C 1.156571 -0.422957 -3.247411
C 2.408092 -0.309964 -3.749204
H 3.021493 -1.226096 -3.890118
C 2.938667 0.929124 -4.052476
C 2.115640 2.034425 -3.891804
C 0.869360 1.896788 -3.384925
H 0.253999 2.808927 -3.227137
H 2.494720 3.046252 -4.153365
N 4.293591 1.209674 -4.434389
```

N 5.091958 0.220358 -4.566411

C 6.474756 0.512166 -4.821228

C 7.324654 -0.583508 -4.870198

C 8.660939 -0.414722 -4.992374

H 9.308445 -1.317952 -4.995494

C 9.228530 0.830901 -5.065739

C 8.380676 1.906904 -5.081777

C 7.041267 1.764585 -4.959540

H 6.395925 2.669203 -4.954612

H 8.802716 2.931824 -5.165350

C 10.367779 0.976503 -5.120630

H 6.905002 -1.609786 -4.788370

H 0.783676 -1.434652 -2.978452

H -1.277564 1.248497 -3.123835

H -1.032556 -0.363715 -2.846298

S -1.501121 0.708065 -0.850141

H -0.163693 0.210951 1.048740

H -0.292510 -1.039075 -0.098885

H 11.486645 0.338729 -2.044173

H 11.445255 1.979995 -1.918387

S 11.896331 1.309652 -4.106367

H 10.467139 1.736194 -5.889344

H 10.706076 0.120271 -5.694966

- Minimum of the $S_0^{(d)}/S_1^{(d)}$ conical intersection at exciton TC level

C 0.000000 0.000000 0.000000

C 1.148184 0.000000 0.000000

C 1.871459 1.156815 0.000000

C 3.231104 1.167061 -0.022701

H 3.770943 2.135601 -0.024788

H 1.332459 2.127249 0.026972

C 1.848638 -1.179814 0.013294

H 1.296675 -2.144125 0.026118

C 3.194386 -1.206746 0.042088

H 3.727248 -2.179909 0.070359

C 3.931407 -0.025695 0.036561

N 5.323661 -0.125728 0.057547

N 6.358352 0.619922 0.016803

C 7.116676 0.961590 -1.118065

C 6.850312 0.605826 -2.436099

C 7.714354 0.937457 -3.423761

H 7.474287 0.609423 -4.457887

H 5.936176 0.022860 -2.673627

C 8.266343 1.712476 -0.875979

H 8.509386 2.030661 0.161407

C 9.091681 2.050878 -1.886648

H 9.996081 2.656603 -1.661236

C 8.855293 1.663661 -3.178397

C 9.637883 1.921622 -3.985437

C -1.316253 1.557152 -1.755321

C -0.447879 1.986778 -2.357653

C 0.234354 1.233916 -3.279014

C 1.318096 1.717822 -3.924856

H 1.860197 1.063545 -4.641694

C 1.779419 2.999764 -3.672673

C 1.029724 3.797643 -2.821267

C -0.051312 3.286128 -2.185755

H -0.606722 3.936291 -1.476688

H 1.344319 4.845719 -2.626704

N 3.030246 3.534552 -4.120925

N 3.698958 2.824150 -4.950998

C 5.035675 3.227026 -5.256970

C 5.695962 2.453232 -6.199962

C 7.016783 2.628286 -6.427118

H 7.514831 1.964365 -7.166363

C 7.745633 3.565957 -5.739111

C 7.071723 4.389751 -4.875952

C 5.751499 4.230206 -4.632262

H 5.250519 4.897439 -3.898546

H 7.630551 5.183033 -4.333990

C 8.872783 3.679540 -5.868241

H 5.142635 1.657104 -6.744500

```
H -0.076827 0.185931 -3.481586
H -2.076057 2.326639 -1.841056
H -1.751409 0.799108 -2.398213
S -1.683306 0.812128 -0.092243
H -0.101217 -0.497088 0.958221
H -0.189991 -0.743858 -0.767950
H 9.482540 1.172158 -4.754430
H 10.530509 1.523716 -3.513830
S 10.465780 3.216099 -5.046024
H 8.981519 4.748023 -6.023519
H 9.070293 3.337765 -6.878699
```

- Ground state *trans*-*trans* minimum at exciton EC level

```
C 0.000000 0.000000 0.000000
C 1.143620 0.000000 0.000000
C 1.806770 1.194312 0.000000
C 3.154952 1.268634 -0.092582
H 3.645245 2.265317 -0.123553
H 1.213623 2.133402 0.039800
C 1.904699 -1.143488 0.014480
H 1.424351 -2.143862 0.073054
C 3.252784 -1.095971 -0.078349
H 3.835148 -2.043086 -0.099729
C 3.915180 0.119354 -0.183144
N 5.321098 0.040958 -0.456213
N 5.959755 1.138028 -0.612201
C 7.333164 1.052419 -1.018511
C 8.060844 -0.103756 -1.228458
C 9.321308 -0.049213 -1.715824
H 9.858551 -1.005141 -1.897122
H 7.604295 -1.094102 -1.014406
C 7.963232 2.260831 -1.273937
H 7.419385 3.213987 -1.095990
C 9.223538 2.289542 -1.765166
H 9.685994 3.275138 -1.989527
C 9.922961 1.142288 -2.030287
```

C 10.975403 1.186765 -2.489594

C -0.660252 0.598956 -2.512088

C 0.372112 0.677007 -3.009395

C 1.156496 -0.423004 -3.247454

C 2.408003 -0.310074 -3.749253

H 3.021353 -1.226230 -3.890184

C 2.938661 0.928973 -4.052522

C 2.115706 2.034320 -3.891849

C 0.869338 1.896743 -3.384929

H 0.253990 2.808882 -3.227130

H 2.494848 3.046126 -4.153404

N 4.293582 1.209385 -4.434435

N 5.091880 0.219934 -4.566441

C 6.474679 0.511568 -4.821243

C 7.324430 -0.584205 -4.870196

C 8.660756 -0.415637 -4.992317

H 9.308119 -1.318978 -4.995418

C 9.228609 0.829885 -5.065630

C 8.380997 1.906096 -5.081654

C 7.041319 1.763911 -4.959584

H 6.396073 2.668599 -4.954686

H 8.803275 2.930924 -5.165178

C 10.367863 0.975261 -5.120458

H 6.904648 -1.610431 -4.788354

H 0.783584 -1.434707 -2.978520

H -1.277574 1.248493 -3.123808

H -1.032603 -0.363737 -2.846304

S -1.501049 0.708011 -0.850099

H -0.163683 0.210976 1.048738

H -0.292516 -1.039077 -0.098860

H 11.486829 0.339549 -2.044332

H 11.445379 1.980820 -1.918572

S 11.896397 1.308094 -4.106067

H 10.467407 1.734947 -5.889148

H 10.706027 0.118973 -5.694781

- Minimum of the $S_0^{(d)}/S_1^{(d)}$ conical intersection at exciton EC level

C 0.000000 0.000000 0.000000

C 1.142967 0.000000 0.000000

C 1.853809 1.162529 0.000000

C 3.207650 1.188403 -0.033028

H 3.732305 2.164806 -0.067677

H 1.306265 2.128711 0.024727

C 1.860330 -1.174831 0.006636

H 1.319772 -2.146783 0.019778

C 3.210277 -1.188246 -0.000946

H 3.753613 -2.156311 0.003775

C 3.921688 0.003044 -0.013798

N 5.317843 -0.032023 0.082316

N 6.308047 0.747603 0.148118

C 7.212853 1.162273 -0.852338

C 7.144151 0.806379 -2.188091

C 8.113365 1.180002 -3.052080

H 8.026914 0.844164 -4.108190

H 6.294459 0.189194 -2.550938

C 8.289878 1.943302 -0.449795

H 8.372631 2.263498 0.611432

C 9.234492 2.316328 -1.339690

H 10.080157 2.948427 -0.991593

C 9.179472 1.952137 -2.661038

C 10.031915 2.257157 -3.371453

C -1.219828 1.605427 -1.772990

C -0.315495 2.039004 -2.325446

C 0.413452 1.304426 -3.226214

C 1.536299 1.795031 -3.796756

H 2.113888 1.155726 -4.498339

C 1.990305 3.064007 -3.486456

C 1.197543 3.843772 -2.658030

C 0.076664 3.332089 -2.101530

H -0.513015 3.967237 -1.405901

H 1.505494 4.884563 -2.417425

N 3.266195 3.615918 -3.837879

N 4.007324 2.943682 -4.635778

C 5.351684 3.398576 -4.838546

C 6.106171 2.675939 -5.750715

C 7.429765 2.911942 -5.891503

H 8.000894 2.285669 -6.610264

C 8.076029 3.860888 -5.141001

C 7.312302 4.626995 -4.300229

C 5.985858 4.411948 -4.144868

H 5.410383 5.045594 -3.436097

H 7.797594 5.431180 -3.706138

C 9.206710 4.035606 -5.210220

H 5.623758 1.876501 -6.353935

H 0.111118 0.262924 -3.468170

H -1.968479 2.382775 -1.883423

H -1.625664 0.861890 -2.450802

S -1.662157 0.835924 -0.137157

H -0.138713 -0.513093 0.944347

H -0.174812 -0.726951 -0.787418

H 10.001160 1.514993 -4.162164

H 10.890795 1.897419 -2.813913

S 10.803210 3.646513 -4.344092

H 9.266980 5.112780 -5.328306

H 9.468934 3.736716 -6.219536
